# Identification of the Unstable Human Postural Control System

**DOI:** 10.3389/fnsys.2016.00022

**Published:** 2016-03-11

**Authors:** Sungjae Hwang, Peter Agada, Tim Kiemel, John J. Jeka

**Affiliations:** ^1^Department of Kinesiology, Temple UniversityPhiladelphia, PA, USA; ^2^Department of Kinesiology, University of MarylandCollege Park, MD, USA; ^3^Department of Bioengineering, Temple UniversityPhiladelphia, PA, USA

**Keywords:** multi-sensory perturbation, sensory reweighting, direct effect, plant, feedback, postural control

## Abstract

Maintaining upright bipedal posture requires a control system that continually adapts to changing environmental conditions, such as different support surfaces. Behavioral changes associated with different support surfaces, such as the predominance of an ankle or hip strategy, is considered to reflect a change in the control strategy. However, tracing such behavioral changes to a specific component in a closed loop control system is challenging. Here we used the joint input–output (JIO) method of closed-loop system identification to identify the musculoskeletal and neural feedback components of the human postural control loop. The goal was to establish changes in the control loop corresponding to behavioral changes observed on different support surfaces. Subjects were simultaneously perturbed by two independent mechanical and two independent sensory perturbations while standing on a normal or short support surface. The results show a dramatic phase reversal between visual input and body kinematics due to the change in surface condition from trunk leads legs to legs lead trunk with increasing frequency of the visual perturbation. Through decomposition of the control loop, we found that behavioral change is not necessarily due to a change in control strategy, but in the case of different support surfaces, is linked to changes in properties of the plant. The JIO method is an important tool to identify the contribution of specific components within a closed loop control system to overall postural behavior and may be useful to devise better treatment of balance disorders.

## Introduction

Human upright standing is intrinsically unstable, requiring sensory feedback to remain upright. The feedback process involves the integration of sensory information from multiple sources (e.g., visual, vestibular, and somatosensory system) that is continually reweighted due to neurological injury or when environmental conditions change (van der Kooij et al., 2001; Peterka, 2002; Kiemel et al., 2008, 2011).

**Figure 1:**
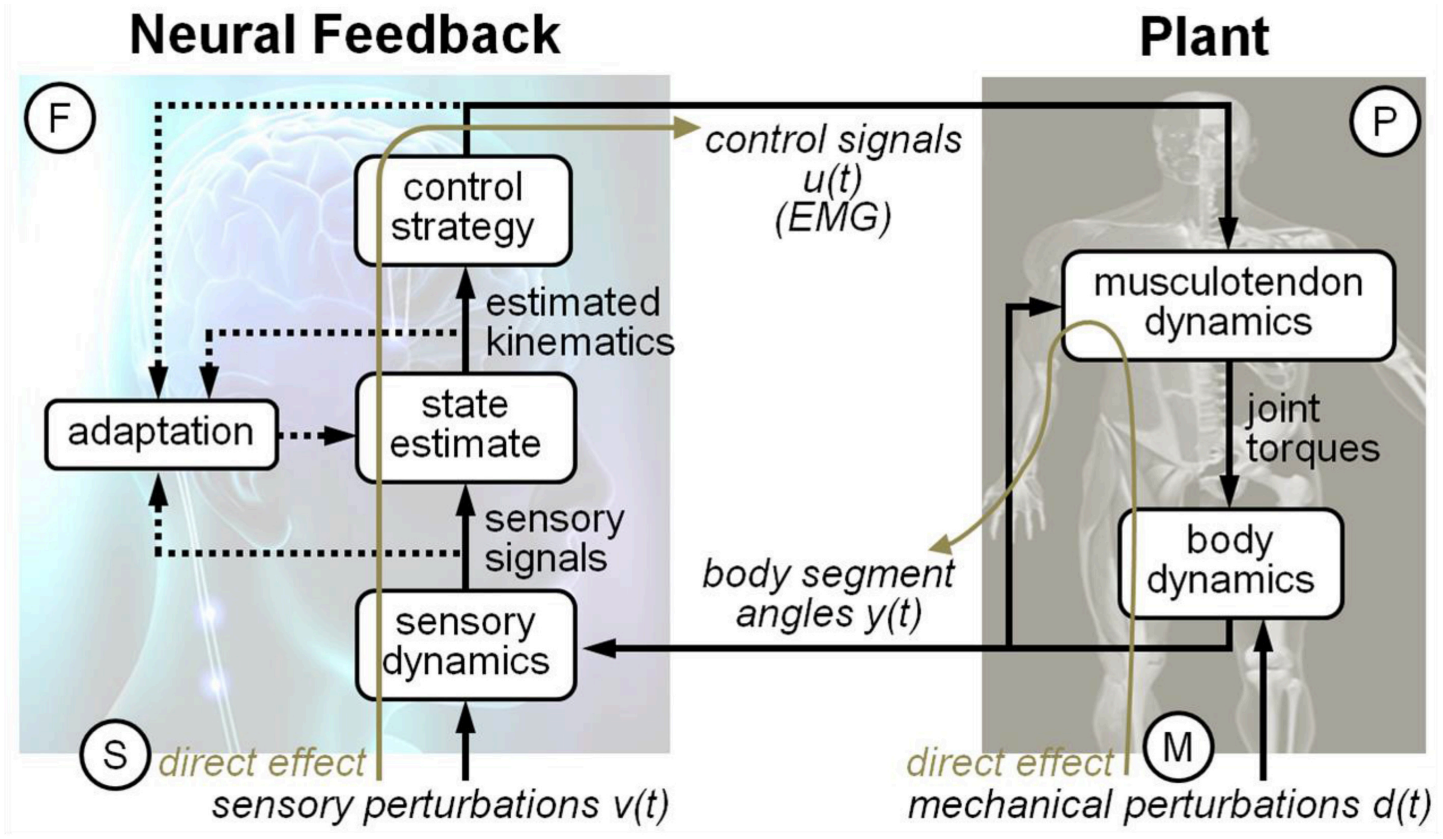
**Schematic diagram of the human postural control feedback loop**. The human postural control feedback loop consists of three major components: feedback, plant, and direct effect.

Our current understanding of the mechanisms underlying postural control is best illustrated through a simple example: the experiments that impose support surface translations on a normal and shortened surface (Horak and Nashner, 1986). On a normal support surface, subjects adopt an “ankle pattern,” in response to small disturbances, whereas on a shortened surface, they adopt a “hip pattern” of control. But what is the mechanistic basis for this pattern switch? How do different components of human postural control loop contribute to this pattern switch? Does it entail a change in “control strategy,” a process within the feedback side of the control loop, or does standing on a shortened surface change the biomechanical properties of the body or its mechanical interaction with the environment (e.g., the mechanical interaction between the feet and the surface changes when the surface is shortened)? Our current understanding of such phenomena is descriptive, not mechanistic. It is a great challenge to trace behavioral deficits to a particular component of the loop, because of the complicated interactions between multiple processes that co-exist, as depicted in Figure 1.

A classic solution to this puzzle is to “open the loop” and study separate components in isolation. Such techniques, for example, were used to study the properties of a deafferented muscle by stimulating the muscle artificially to mimic motor commands (Houk and Rymer, 1981). Open-loop techniques have also proven effective in behaviors such as eye movements (Leigh et al., 1982; Stark, 1984). So why not apply such techniques to postural control? Because the bipedal body standing on a surface (i.e., the plant) is inherently unstable, if all sensory input is removed, standing upright is no longer possible. For example, individuals with bilateral vestibular loss cannot stay upright in Condition 6 of the Sensory Organization Test, which removes visual and proprioceptive input through sway-referencing (Peterka and Black, 1990). To solve this problem, we recently implemented an approach called the joint input–output (JIO) method (e.g., Fitzpatrick et al., 1996; Katayama, 2005; van der Kooij et al., 2005; Kiemel et al., 2008, 2011). The JIO method “opens the loop” for human upright standing by calculating multiple linear input–output relationships (gain and phase), between various components of the control loop and then using mathematical techniques to isolate a particular process (e.g., feedback, see Materials and Methods Section below). The JIO method has been also applied in clinical studies (Engelhart et al., 2014; Pasma et al., 2014). For example, van der Kooij's group used the JIO method in order to detect asymmetries in balance control of Parkinson patients with system identification (van der Kooij et al., 2007; Boonstra et al., 2014). In Pasma's recent review paper, they argue that as current clinical balance tests only measure the ability to maintain standing balance and cannot distinguish between cause and effect in a closed loop, there is a clear clinical need for new techniques to assess standing balance. A way to disentangle cause-and-effect relations to identify primary defects and compensation strategies is based on the application of external disturbances and system identification techniques, applicable in clinical practice (Pasma et al., 2014).

In this study, our goal was to understand how different components of the control loop contribute to the flexible control of upright stance on different support surfaces. We attempted to decode the mechanistic basis behind any changes in body movements and muscle activations on a normal support surface in contrast to a short support surface using the JIO system identification method. The results from the decomposition of human postural control loop show a dramatic phase reversal between visual input and body kinematics due to the change in surface condition from trunk leads legs to legs lead trunk as frequency of visual perturbation increases. Importantly, we are able to definitively attribute this change to two different processes, one in the plant and one in the feedback portion of the control loop. Considering the many subsystems/processes involved in human upright stance control, such findings may have implications for populations with poor balance control, fostering more precise diagnosis, and treatment.

## Materials and methods

### Joint input–output (JIO) method

The classical view of human upright standing is a control system consisting of a plant and a feedback (i.e., neural control) operating continuously in a closed loop, as shown in Figure 1. We applied the JIO method for human postural control using independent sensory perturbations to identify the plant and independent mechanical perturbations to identify the feedback (Kiemel et al., 2008, 2011). Figure 1 traces the path from input-to-output of a visual perturbation, which generates a number of closed loop frequency response functions (FRFs): EMG (ankle and hip) responses relative to the visual motion and kinematic (trunk and leg) responses relative to visual motion. Closed-loop FRFs reflect the interaction between the plant and the feedback components of the control loop, as well as properties of the perturbation. Such interactions make closed-loop FRFs notoriously difficult to interpret mechanistically. JIO method, however, states that the relationship between two closed-loop FRFs depends only on either the plant or the feedback. For example, dividing the FRF from visual perturbations to trunk/leg angles by the FRF from visual perturbations to the EMG signals results in an open-loop FRF from ankle/hip muscular activity to trunk/leg angles, that is, an open-loop FRF that reflects only properties of the plant. Essentially the feedback component is canceled by the ratio of the two closed-loop FRFs because it is common to both closed-loop FRFs. A similar approach defines properties of neural feedback through FRFs generated by mechanical perturbations. An important aspect of the JIO method is that to completely identify the control loop of the multi-segmented body, the number of perturbations must match the number of inputs to the plant or feedback. Thus, the number of sensory perturbations must equal the number of EMG inputs (ankle and hip) to the plant and the number of mechanical perturbations must equal the number of kinematic inputs (trunk and leg) to feedback. In additional, the JIO-method formulas in Section Identification of the Direct Effects, the Plant, and the Feedback Frequency Response Function (FRF) below are based on the assumption that all perturbation signals are mutually uncorrelated, which we ensure by designing signals that are mutually statistically independent.

### Experimental methods

Twenty healthy subjects (10 males, 10 females, age: 21.5 ± 2.5 years, height: 171.6 ± 12.3 cm, weight: 68.8 ± 17.4 kg, foot length: 250.3 ± 2.9 mm) who were students in University of Maryland were participated in this experiment. All the subjects were physically healthy and active, with no known musculoskeletal injuries, or neurological disorders that might affect their ability to maintain balance. The Institutional Review Board at the University of Maryland approved the experimental protocol. All the subjects received oral and written instructions for the experiment procedures and gave informed written consent according to guidelines implemented by the Institutional Review Board at the University of Maryland before undertaking the experiment.

**Figure 2:**
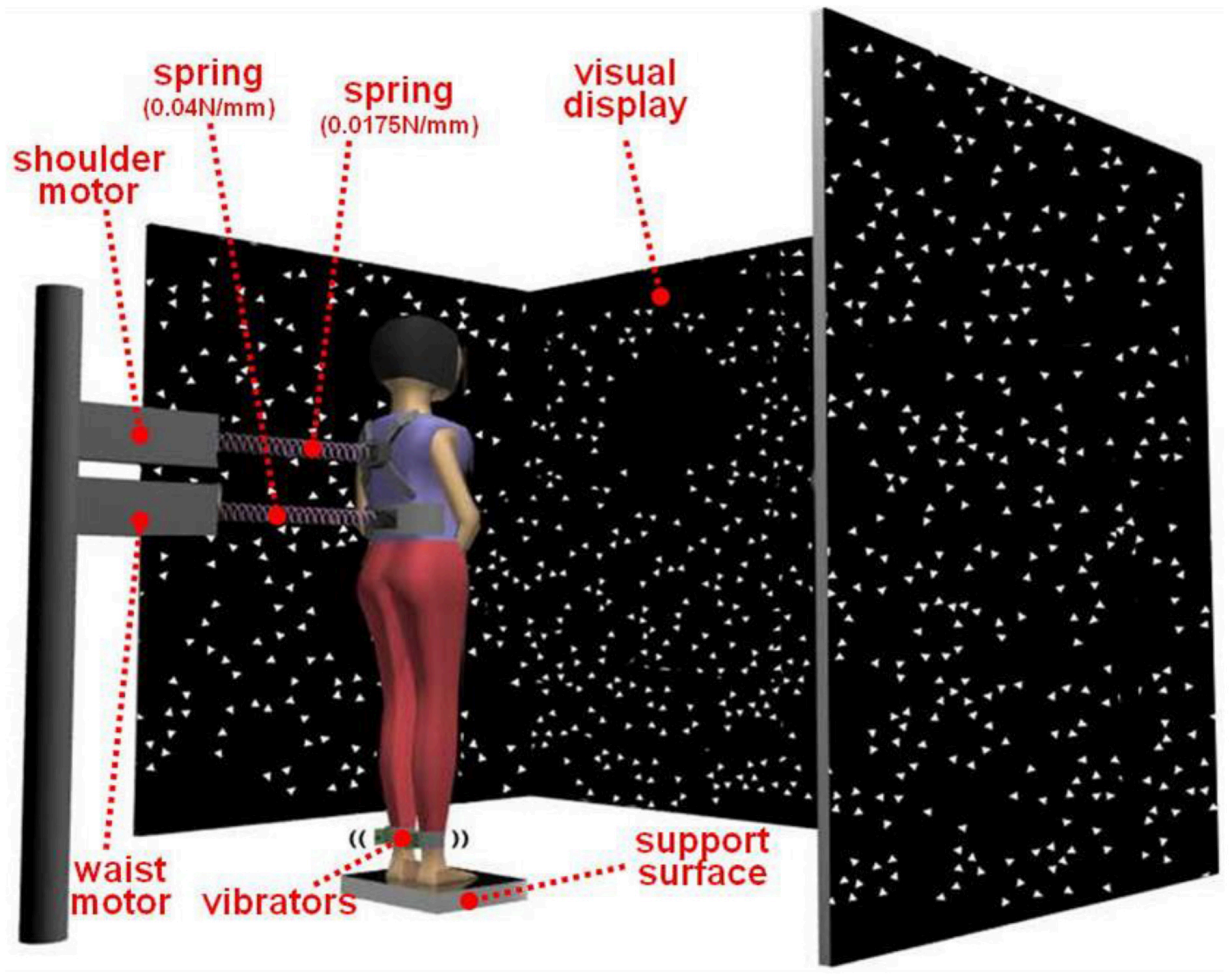
**Experimental setup for this study**. Two mechanical perturbations (waist and shoulder perturbation) and two sensory perturbations (visual and proprioceptive perturbations) were applied to the subject during standing. Subjects stood on different two width (normal or short) support surface in the middle of the visual cave and faced the front wall.

Figure 2 shows the experimental setup for this study. Two independent mechanical perturbations (waist and shoulder perturbation) and two independent sensory perturbations (visual and proprioceptive perturbations) were applied to the subject during standing. Subjects stood on different two width (normal or short) support surface in the middle of the visual cave and faced the front wall. Subjects assumed a foot position with heels at a distance of ~11% of their heights and an angle of 14° between each foot and the midline (McIlroy and Maki, 1997). And each subject's foot position was marked to keep same foot position during the experiment. The instruction to the subjects was to look straight ahead at the front screen and not to focus on any particular triangle. In addition, subjects were instructed to maintain a comfortable upright stance with folded hand in front of body and not to consciously resist any force from the mechanical perturbations. There were two conditions with all the perturbations applied simultaneously. One condition is standing on a normal support surface and another condition is standing on a short support surface (50% foot length) with no support under the balls and toes of the feet. The order of condition was randomized. The length of each trial was 240 s. Another 5 s in the beginning and at the end were added to allow the motors to speed up or slow down. Five repetitions were run in each condition for each subject.

Kinematics were captured by Vicon MX digital optical motion capture system with five infrared cameras (Vicon, UK). The shoulder (the scapula), hip (the greater trochanter), knee (the lateral femoral condyle), ankle (the lateral malleolus), and foot (the first metatarsal head) were measured by attaching five reflective markers on the right side of the subject to measure subject's anterior-posterior (AP) movement in the sagittal plane. Kinematics were sampled at 300 Hz. Muscle activity was measured using multi-channel telemetric surface EMG systems (ZeroWire, Aurion, and Trigno Wireless System, Delsys). Wireless electrodes were placed on the right side of the body measuring 11 muscles: lateral gastrocnemius, medial gastrocnemius, soleus, tibialis anterior are for ankle EMG signals and biceps femoris, semitendinosus, rectus femoris, vastus lateralis, vastus medialis, rectus abdominus, and erector spinae muscles of the lumbar spine for hip/lower trunk EMG signals. EMG signals were sampled at 2400 Hz.

In order to have uncorrelated signals when applying all perturbations simultaneously, we used filtered white noise generated with two low-pass Butterworth filters for the mechanical perturbations: a first-order filter and an eighth-order filter with cutoff frequencies of 0.1 and 5 Hz, respectively. The power spectrum density (PSD) of the white noise was 4 and 2.5 cm^2^/Hz, respectively, for the waist and shoulder perturbations. We used different seeds for every trial and subject. The resulting peak-to-peak amplitude for the waist motor and shoulder motor displacement was 13–15 and 11.5–13.5 cm, respectively. The same procedure was used for the visual signal except that the PSD was 4.05 deg^2^/Hz and the cutoff frequency of the first filter was 0.02 Hz. The mechanical perturbations were delivered through two linear motors (LX80L, Parker Hannifin Corporation, USA), which were positioned behind the subjects and pulled subjects from their backs. The actual displacements of the motors were used as mechanical perturbation signals. Weak and continuous mechanical perturbations were applied to the subject by attaching springs to a waist belt and shoulder-strap worn by the subject on one end and computer controlled linear motors on the other. The spring constants for the waist belt and shoulder-strap were 0.04 and 0.0157 N/mm, respectively.

The visual display was projected by JVC projectors (Model: DLA-M15U, Victor Company, Japan) to three mirrors, which reflected and rear-projected onto a visual cave consisting of three 2.67 × 3.33 m screens (Fakespace, Inc., Marshalltown, Iowa, USA). The visual display consisted of 500 randomly distributed white triangles (3.4 × 3.4 × 3 cm) on a black background. To reduce aliasing effects in the foveal region, no triangles were displayed within a horizontal band of ±5° at eye height. The frame rate of the visual display was 60 Hz. A visual signal was displayed as a visual rotation around ankle joint. Bilateral vibration of Achilles tendons was applied through two 20 mm vibrator motors, driven at 80 Hz and 1 mm amplitude displacement. The vibrators are enclosed in a hollow rectangular PVC container (3.5 × 3.8 × 3.5 cm) with a flexible recessed surface mounted on the contact face for comfortable fitting around the Achilles tendon. The enclosure was held in place by an elastic strap. The vibrators were randomly turned on and off by the control signal. Three reflective markers attached on triangular plane which was connected with each PVC container to measure vibrations of the vibrator during standing.

### Signal processing

**Figure 3:**
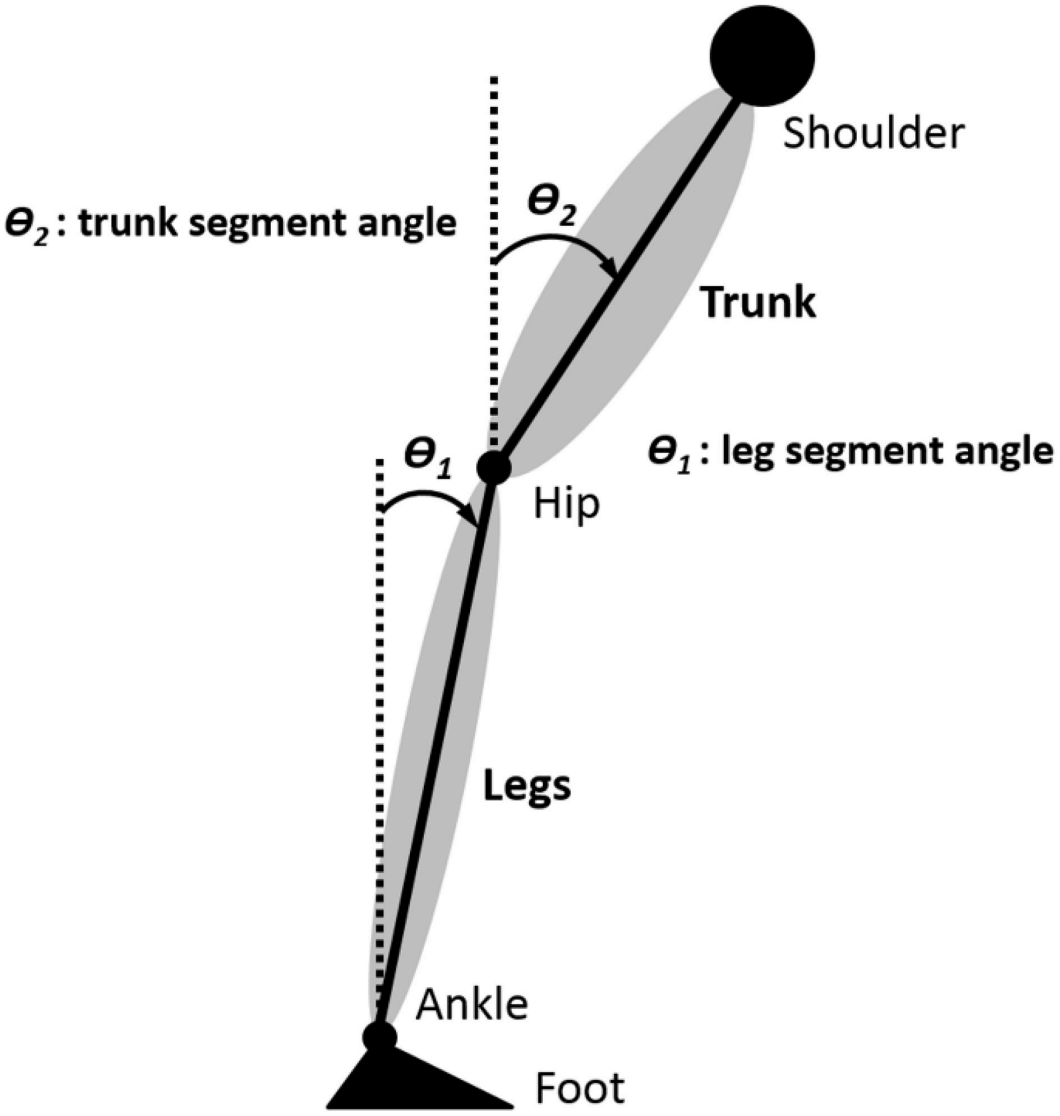
**Definition of body segment angles**. The leg angle θ_1_(*t*) and trunk angle θ_2_(*t*) with respect to vertical were determined by the anterior-posterior (AP) and vertical displacement of the shoulder, hip, ankle marker, and foot marker.

We approximated the body as having two mechanical degrees of freedom in the sagittal plane (see Discussion). The leg angle θ_1_(*t*) and trunk angle θ_2_(*t*) with respect to vertical were determined by the AP and vertical displacement of the shoulder, hip, ankle marker, and foot marker, as shown in Figure 3. For the EMG activity of each muscle, the mean was subtracted from the raw EMG and then the raw EMG was rectified, resulting in ankle EMG signals *u*_11_(*t*), *u*_12_(*t*), *u*_13_(*t*), and *u*_14_(*t*) and hip/lower-trunk EMG signals *u*_21_(*t*), *u*_22_(*t*), …*, u*_27_(*t*) (i.e., *u*_*ij*_(*t*) was defined as *i* = 1 for the ankle EMG signal and *j* = 1, 2, 3, 4 for four ankle EMG signals. Also, *i* = 2 for the hip/lower-trunk EMG signal and *j* = 1, 2, 3, 4, 5, 6, 7 for seven hip/lower-trunk EMG signals).

For any two signals *x*(*t*) and *y*(*t*), the power spectral densities (PSDs) *p*_*xx*_(*f*) and *p*_*yy*_(*f*) and cross spectral density (CSD) *p*_*xy*_(*f*), where f is frequency, were computed using Welch's method with 40-s Hanning windows and 50% overlap and then averaged across trials (Bendat and Piersol, 2000). Complex coherence is $c_{xy}\left(f\right)=p_{xy}\left(f\right)∕\sqrt{p_{xx}\left(f\right)p_{yy}\left(f\right)}$ and (magnitude-squared) coherence is ${\left|c_{xy}\left(f\right)\right|}^{2}$. Coherence is a measure of the strength of the linear relationship between *x* and *y*.

The closed-loop frequency response function (FRF) from *x*(*t*) to *y*(*t*) is *H*_*xy*_(*f*) = *p*_*xy*_(*f*)/*p*_*xx*_(*f*). Gain is the absolute value of *H*_*xy*_(*f*) and phase is the argument of *H*_*xy*_(*f*), converted to degrees. A positive phase indicates that *y*(*t*) was phase advanced relative to *x*(*t*). Following Kiemel et al. (2008), we defined the *coherence-weighted mean FRF* across subjects as ${\bar{H}}_{xy}\left(f\right)={\bar{c}}_{xy}\left(f\right)\sqrt{{\bar{p}}_{yy}\left(f\right)∕{\bar{p}}_{xx}\left(f\right)}$, where ${\bar{c}}_{xy}\left(f\right)$ is the mean complex coherence and ${\bar{p}}_{xx}\left(f\right)$ and ${\bar{p}}_{yy}\left(f\right)$ are the geometric mean PSDs.

The four ankle EMG signals were used to compute the *weighted ankle EMG signal u*_1_(*t*) = *w*_11_*u*_11_(*t*) + *w*_12_*u*_12_(*t*) + *w*_13_*u*_13_(*t*) + *w*_14_*u*_14_(*t*). Weights were adjusted using the Matlab optimization toolbox to maximize the average coherence between the four perturbation signals and *u*_1_(*t*) for frequencies from 0.025 to 5 Hz subject to the constraints that *w*_1*j*_ ≥ 0 for posterior muscles, *w*_1**j**_ ≤ 0 for anterior muscles, and |*w*_1_| + |*w*_2_| + |*w*_3_| + |*w*_4_| = 1 Similarly, the seven hip and lower-trunk EMG signals were used to define the *weighted hip EMG signal u*_2_(*t*) = *w*_21_*u*_21_(*t*) + *w*_22_*u*_22_(*t*) + ⋯ + *w*_27_*u*_27_(*t*). Each weighted EMG signal was normalized by dividing by the square root of its power from 0 to 5 Hz, calculated by integrating its PSD.

Use of a 40-s spectral window resulted in PSDs and CSDs computed at 200 frequencies from 0.025 to 5 Hz. To increase statistical power, frequencies were divided into 10 frequency bins and PSD and CSD values were averaged within each bin. Frequency binning was done after finishing EMG normalization and weighting, and before calculating FRFs. The basic principle was trying to get 10 equal-spaced bins in a log scale with additional fine adjustments to ensure that responses were significantly different from zero. The average frequencies for each bin were:

$$\begin{array}{l} {}[0.062,0.125,0.2375,0.425,0.637,0.9625,1.4375,1.8875,\\ \;3.1625,4.3875]. \end{array}$$

In order to understand the dynamic relationship between trunk and leg segments (or hip muscles and ankle muscles) during standing on normal support surface and short support surface with multiple sensory and mechanical perturbation, we calculated cophase from the complex coherence between the trunk and legs (or the weighted hip EMG and weighted ankle EMG). The trunk (or weighted hip EMG) was used as the reference, so positive cophase means legs leading trunk (or ankle muscles leading hip muscles).

### Identification of the direct effects, the plant, and the feedback frequency response function (FRF)

We applied the JIO method of closed-loop system identification to postural control in the sagittal plane (Katayama, 2005; van der Kooij et al., 2005). Previous applications of the JIO method to postural control have focused on the plant and/or feedback (e.g., Fitzpatrick et al., 1996; Kiemel et al., 2008, 2011; Boonstra et al., 2013; Engelhart et al., 2015). In this study, we proposed to identify the direct effects of sensory and mechanical perturbations in addition to the plant and feedback based on the theoretical framework of Figure 1. The direct sensory effect is the effect that sensory perturbations would have on EMG signals in a (hypothetical) open-loop condition in which EMG signals no longer affect kinematic variables. The direct mechanical effect is the effect that mechanical perturbations would have on kinematic variables in a (hypothetical) open-loop condition in which kinematic variables no long affect EMG signals.

We assumed a linear approximation of each process within each experimental condition of standing on the normal support surface and standing on the short support surface respectively. We carried out JIO method separately for each condition, to account for non-linearities such as sensory reweighting. Let *u(t)* be a *n*_*u*_-by-1 vector of weighted EMG signals, *y(t)* be a *n*_*y*_-by-1 vector of body segment angles, *v(t)* be a *n*_*v*_-by-1 vector of sensory perturbations, and *d(t)* be a *n*_*d*_-by-1 vector of

(1a) $$\begin{array}{r} Y\left(f\right)=P\left(f\right)U\left(f\right)+M\left(f\right)D\left(f\right)+N_{P}\left(f\right), \end{array}$$

(1b) $$\begin{array}{r} U\left(f\right)=F\left(f\right)Y\left(f\right)+S\left(f\right)V\left(f\right)+N_{F}\left(f\right), \end{array}$$

where *P(f)* is the open-loop FRF of the plant, *M(f)* is an open-loop FRF characterizing the direct effect of the mechanical perturbations on body segment angles, *N*_*P*_(*f*) is the Fourier transform of intrinsic noise in the plant, *F(f)* is the open-loop FRF of the feedback, *S(f)* is an open-loop FRF characterizing the direct effect of the sensory perturbations on the EMG signals, *N*_*F*_(*f*) is the Fourier transform of intrinsic noise in the feedback. Since the plant and feedback are multiple-input, multiple-output (MIMO), *P(f), F(f), S(f)*, and *M(f)* are matrices for each frequency *f*. The key to the JIO method is that it uses the relationships among closed-loop FRFs to identify the plant, feedback and direct effects. Denoting the closed-loop FRF from *a(t)* to *b(t)* by *H*_*ab*_(*f*), we can then use equations (1a) and (1b) to express the relationship among closed-loop FRFs as:

(2) $$\begin{array}{r} H_{vy}\left(f\right)=P\left(f\right)H_{vu}\left(f\right),H_{vu}\left(f\right)=F\left(f\right)H_{vy}\left(f\right)+S\left(f\right), \end{array}$$

(3) $$\begin{array}{r} H_{du}\left(f\right)=F\left(f\right)H_{dy}\left(f\right),H_{dy}\left(f\right)=P\left(f\right)H_{du}\left(f\right)+M\left(f\right). \end{array}$$

Here, we have taken advantage of our choice to make sensory and mechanical perturbations uncorrelated with each other and with the noise terms. If the number of sensory perturbations equals the number of EMG inputs to the plant (*n*_*v*_ = *n*_*u*_) and the number of mechanical perturbations equals the number of kinematic inputs to feedback (*n*_*d*_ = *n*_*y*_), then *H*_*vu*_(*f*) and *H*_*dy*_(*f*) are square matrices. If the effects of different perturbations are linearly independent so that *H*_*vu*_(*f*) and *H*_*dy*_(*f*) are invertible at each frequency *f*, then we can identify all four open-loop FRFs non-parametrically (without using a model) as:

(4) $$\begin{array}{r} P\left(f\right)=H_{vy}\left(f\right)H_{vu}{\left(f\right)}^{-1},S\left(f\right)=H_{vu}\left(f\right)-F\left(f\right)H_{vy}\left(f\right), \end{array}$$

(5) $$\begin{array}{r} F\left(f\right)=H_{du}\left(f\right)H_{dy}{\left(f\right)}^{-1},M\left(f\right)=H_{dy}\left(f\right)-P\left(f\right)H_{du}\left(f\right). \end{array}$$

In additional to these open-loop FRFs, in what follows we also focus on the closed-loop FRF *H*_*vy*_(*f*) from sensory perturbations to kinematic signals. From equations (2) we have that,

(6) $$\begin{array}{r} H_{vy}\left(f\right)={\left[I-P\left(f\right)F\left(f\right)\right]}^{-1}\;P\left(f\right)S\left(f\right), \end{array}$$

where *I* is the 2-by-2 identify matrix. Note that Equation (4) is linear in *S*(*f*), implying that *H*_*vy*_(*f*) can be written as the sum

(7) $$\begin{array}{r} H_{vy}\left(f\right)=H_{vy}^{\text{ankle}}\left(f\right)+H_{vy}^{\text{hip}}\left(f\right), \end{array}$$

where $H_{vy}^{\text{ankle}}\left(f\right)$ and $H_{vy}^{\text{hip}}\left(f\right)$ are the effects of the sensory perturbations on kinematics due to their effects on ankle EMG and hip EMG, respectively.

### Statistical analysis

We computed bootstrap standard errors (SEs) for the log gain and phase of all FRFs [*H*_*vy*_(*f*), *H*_*vu*_(*f*), *H*_*du*_(*f*), *H*_*dy*_(*f*), *P(f), F(f), S*(*f*), *M*(*f*)], using 1000 bootstrap resamples. In addition, we also computed bootstrap 95% confidence intervals (CIs) for the surface condition effect as well as the condition-by-output interaction in log-gain and phase of each FRF using the percentile-*t* method with 10,000 bootstrap resamples and 1000 nested bootstrap resamples for variance estimation (Hall, 1988; Zoubir and Boashash, 1998).

## Results

### Phase relationship during standing on different support surface

**Figure 4:**
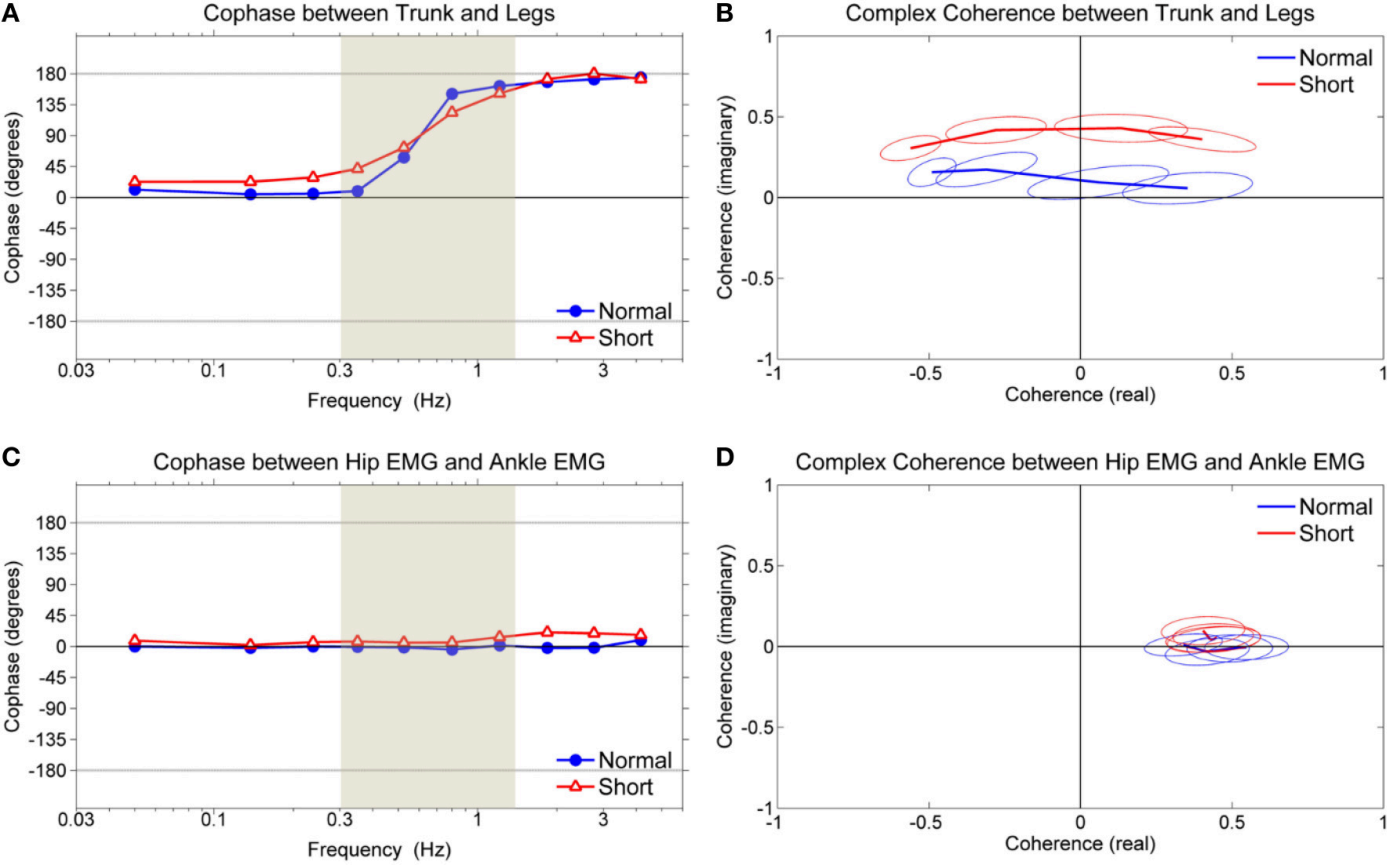
**Cophase and complex coherence. (A)** Cophase between trunk and leg segments, **(B)** complex coherence of shaded region in **(A)** between trunk and leg segments, **(C)** cophase between weighted hip EMG and weighted ankle EMG, **(D)** complex coherence of shaded region in **(C)** between weighted hip EMG and weighted ankle EMG. Ellipses of complex plane represent 95% confidence region in the complex plane.

Figure 4 shows the dynamic relationship between trunk and leg segments (or hip muscles and ankle muscles) during standing on normal support surface and short support surface with multiple sensory and mechanical perturbations.

Figure 4A shows the cophase, which represents the phase angle between the trunk and legs. The cophase for both conditions was ~0° (in-phase) for lower frequencies (below ~0.4 Hz) and ~180° (anti-phase) for high frequencies (above ~1.1 Hz). The in-phase and anti-phase relationships are indicative of the ankle and hip patterns, respectively, demonstrating the simultaneous existence of these patterns during standing on both normal and short support surface (Creath et al., 2005). Both conditions demonstrated a shift from in-phase to anti-phase as frequency increased in an upward direction, suggesting a legs-leading shift. The shift from in-phase to anti-phase was more gradual while standing on short support surface.

Figure 4B shows the complex coherence corresponding to the frequency range defined by the shaded region shown in Figure 4A, illustrating how the coordinative relationship between the trunk and legs changes in the complex plane. Complex coherence values that lie along the positive real axis represent the in-phase relationship between trunk and legs while values that lie along the negative real axis represent the anti-phase relationship. Likewise, complex coherence values with imaginary components that are >0 represent a “legs-leading” coordinative relationship (the trunk was used as the reference in the calculation of complex coherence) while complex coherence values with imaginary components that are < 0 represent a “trunk-leading” coordinative pattern. Complex coherence in both conditions was above the real axis (i.e., the imaginary part is >0) indicating that the legs are leading the trunk while shifting from in-phase to anti-phase.

In contrast to the trunk/leg relationship, the temporal relationship between hip and ankle muscles was in-phase across all frequencies, as shown in Figure 4C. In Figure 4D, the in-phase relationship corresponding to the frequency range defined by the shaded region shown in Figure 4C. Such results indicate that both hip and ankle muscles activate together regardless of the support surface condition and there was no change in the ankle/hip muscle relationship corresponding to the change in the trunk/leg coordinative relationship shown in Figure 4A.

### Decomposition of human postural control loop

**Figure 5:**
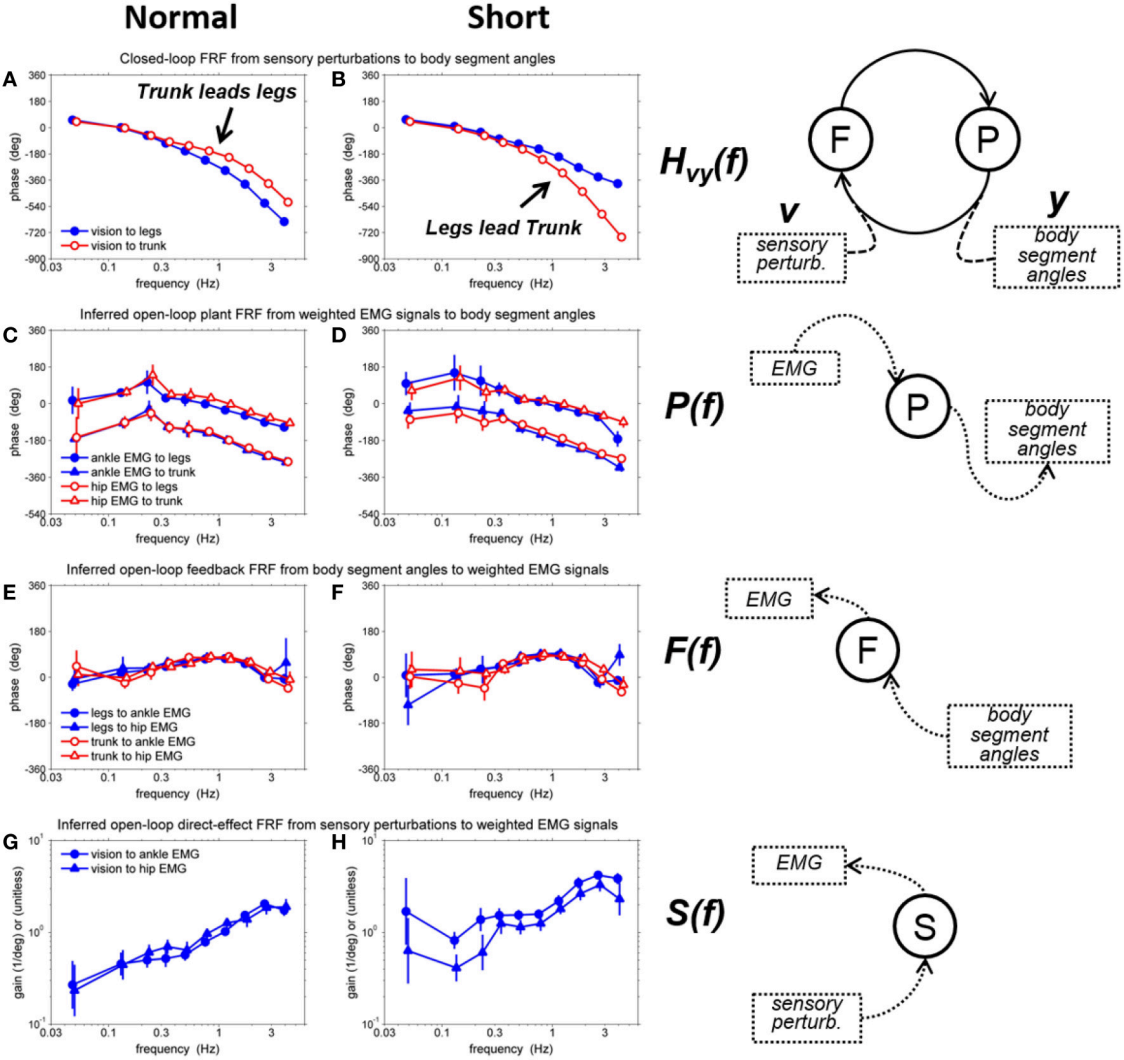
**Representative FRFs on a normal and short support surface. (A)** phase of closed-loop FRF from visual sensory perturbation to body segment angels on the normal support surface, **(B)** phase of closed-loop FRF from visual sensory perturbation to body segment angels on the short support surface, **(C)** phase of inferred open-loop plant FRF from weighted EMG signals to body segment angles on the normal support surface, **(D)** phase of inferred open-loop plant FRF from weighted EMG signals to body segment angles on the short support surface, **(E)** phase of inferred open-loop feedback FRF from body segment angles to weighted EMG signals on the normal support surface, **(F)** phase of inferred open-loop feedback FRF from body segment angles to weighted EMG signals on the short support surface, **(G)** gain of inferred open-loop direct-FRF from visual sensory perturbations to weighted EMG signals on the normal support surface **(H)** gain of inferred open-loop direct-FRF from visual sensory perturbations to weighted EMG signals on the short support surface. Error bars denote bootstrap standard errors.

In order to decode the mechanistic basis underlying the changes in the coordinative relationship between the trunk/leg segments on different support surfaces, we identified all four closed-loop FRFs [*H*_*vu*_(*f*), *H*_*vy*_(*f*), *H*_*du*_(*f*), and *H*_*dy*_(*f*)] and four open-loop FRFs [*P(f), F(f), S(f)*, and *M(f)*] by using a MIMO system identification method. The critical results are the phase relationships of *H*_*vy*_(*f*), *P(f), F(f)*, and the gains of *S(f)*, shown in Figure 5. Error bars denote bootstrap standard errors. Detail descriptions and results of all FRFs are included in the Supplementary Material.

The most remarkable difference between the normal support surface and the short support surface occurred in the phase of the closed-loop FRF, *H*_*vy*_(*f*), between the visual perturbation and the leg and trunk segment angles (Figures 5A,B). On the normal support surface, as the frequency of the visual perturbation increases, larger negative phase values indicate that the legs' response begins to lag behind the trunk's response (i.e., trunk leads legs), as shown in Figure 5A. In contrast, on the short support surface it is the trunk that lags behind the legs (i.e., legs lead trunk), as shown in Figure 5B.

This condition difference in the phase of the closed loop could be due to any combination of three factors. First, the plant may be different between the two conditions, that is, the same muscle activation pattern may produce a different kinematic response because of the resulting mechanical forces imposed while standing on a short vs. a normal surface. Second, feedback (i.e., neural feedback component of the control loop) may be different, because, for example, the nervous system adopts a different control strategy on a short surface. Third, the direct effect of the visual perturbation may be different, because the nervous system may re-weight the use of visual information on a short support surface.

### Effects of each component of human postural control loop on condition difference

**Figure 6:**
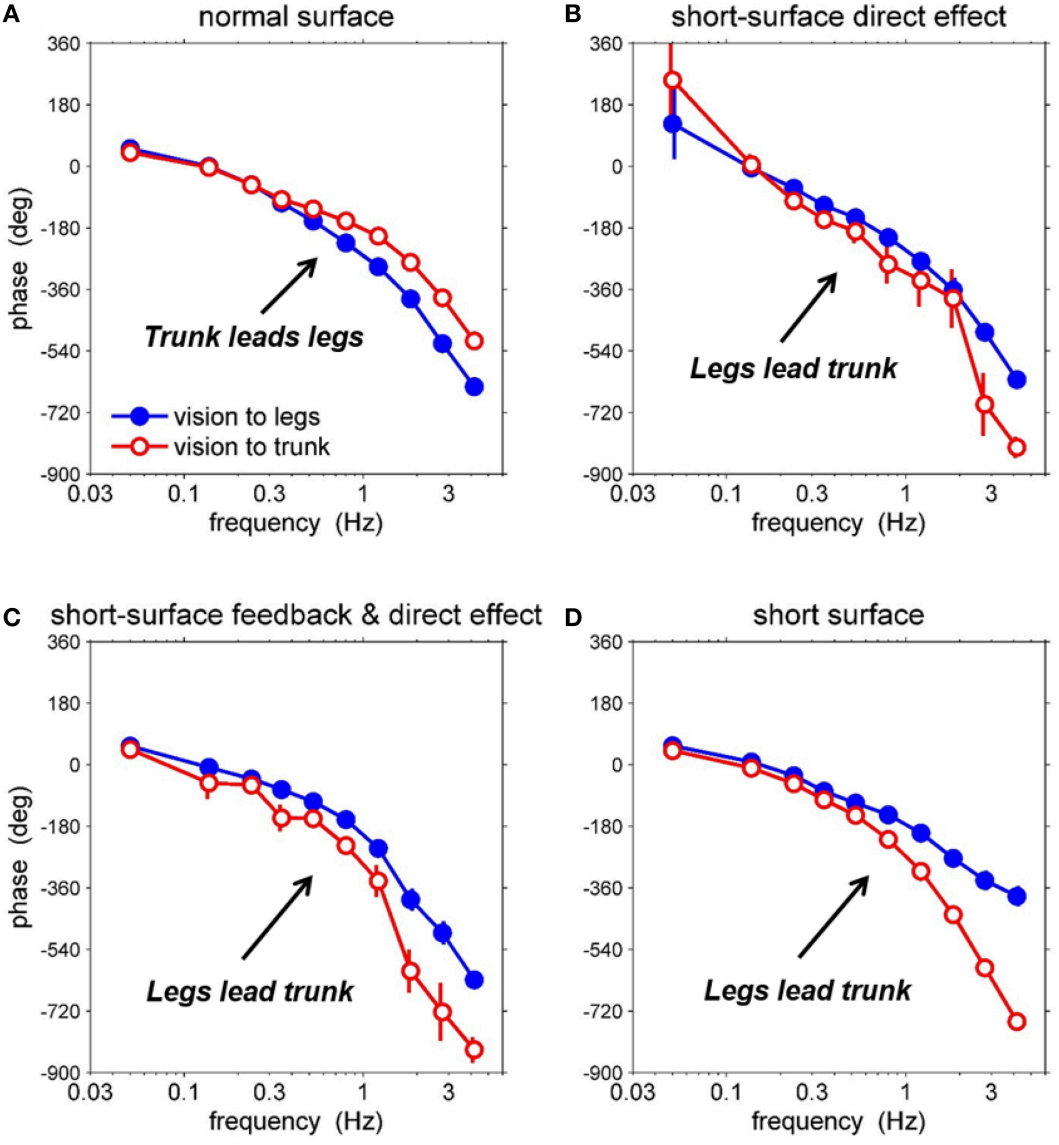
**Effects of short support surface relative to direct effect, feedback, and plant FRFs. (A)** closed-loop FRF of leg/trunk relative to vision on normal supporting surface, **(B)** adding direct effect of short support surface, **(C)** adding feedback and direct effect of short support surface, **(D)** adding plant, feedback, and direct effect of short support surface (i.e., the closed-loop FRF from vision perturbation to body segment angles on short support surface).

Because we were able to separately identify the plant, feedback and direct sensory effect, we can evaluate the effects of different combinations of each component, as illustrated in Figure 6. Figure 6A shows the closed-loop phase response of the legs and trunk to the visual perturbation on the normal support surface, the same results as in Figures 5A, 6B shows a hypothetical case in which the plant and feedback for the normal support surface (Figures 5C,E, respectively) are combined with the direct sensory effect for the short support surface (Figure 5H). Note, that the change in the direct effect by itself increases the phase lag of the trunk at high frequencies so that the trunk now lags behind the legs. Figure 6C shows a second hypothetical case in which the plant for the normal support surface (Figure 5C) is combined with the feedback and direct sensory effect for the short support surface (Figures 5F,H, respectively). The difference in feedback between Figures 6B,C leads to a relatively minor difference in the closed-loop response. Finally, Figure 6D shows the closed-loop response on the short support surface, the same as that in Figure 5B. The change in the plant from Figures 6C,D decreases the phase lag of the legs at high frequencies, further increasing the lag of the trunk behind the legs.

Thus, our analysis suggests that condition differences in both the direct sensory effect and the plant contribute to the condition differences seen in the closed-loop responses of the legs and trunk to movement of the visual scene. These condition differences are not obvious from the closed loop responses alone. Differences in the plant necessarily occur when the mechanical interaction between the feet and the support surface changes. On the other hand, condition differences in the direct sensory effect suggest the existence of an adaptive process such as sensory reweighting.

### Differential sensory reweighting of the direct effect in human postural control loop

**Figure 7:**
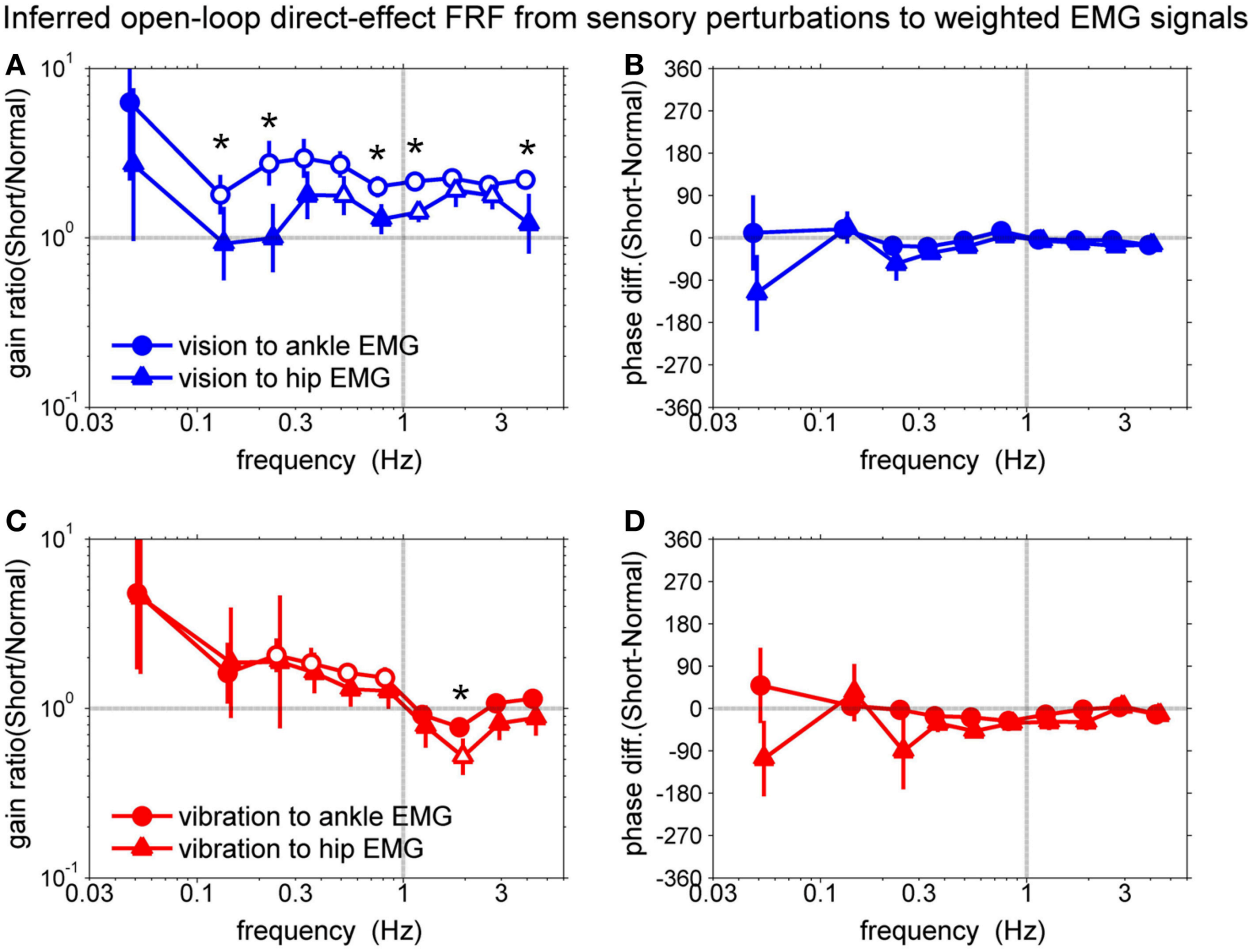
**Comparison of the open-loop direct-effect FRF from sensory perturbations to weighted EMG signals**. **(A)** gain ratio of direct-effect FRFs from vision to EMGs, **(B)** phase difference of direct-effect FRFs from vision to EMGs, **(C)** gain ratio of direct-effect FRFs from vibration to EMGs, **(D**) phase difference of direct-effect FRFs from vibration to EMGs. Open symbols represent significant differences between surface conditions and asterisks represent significant differences between two FRFs based on the bootstrap 95% CIs.

To confirm the sensory reweighting hypothesis, we calculated the gain ratio and phase difference of the FRFs of the direct sensory effect as shown in Figure 7. The gain ratio was calculated from the direct-effect FRF of the short support surface divided by the direct-effect FRF of the normal support surface. The phase difference was calculated from the direct-effect FRF of the short support surface minus the direct-effect FRF of the normal support surface.

Figures 7A,B show the comparison between the direct-effect FRF from the vision sensory perturbation to the weighted ankle EMG signal and the direct-effect FRF from the vision sensory perturbation to the weighted hip EMG signal. Open symbols represent significant differences between surface conditions and asterisks represents significant differences between two FRFs as a condition-by-output interaction (i.e., vision to ankle EMG vs. hip EMG or vibration to ankle EMG vs. hip EMG) based on the bootstrap 95% confidence intervals (CIs).

The gain ratio of the direct-effect FRF from the vision sensory perturbation to the ankle EMG from 2nd to 10th frequency bin was higher than 1. The gain ratio of the direct-effect FRF from the vision sensory perturbation to hip EMG of 5th, 7th, 8th, and 9th frequency bin was higher than 1. This means subjects upweighted vision for ankle muscles and hip muscles during standing on the short support surface. In addition, there are significant differences between two FRFs at 2nd, 3rd, 6th, 7th, and 10th frequency bins (*p* = 0.05, *p* = 0.03, *p* = 0.002, *p* = 0.002, and *p* = 0.02, respectively). In addition, gain ratios for the direct effect FRF for ankle EMG relative to the visual perturbation compared to the hip EMG across 10 frequency bins were significantly higher (*p* = 0.003), indicating that visual upweighting was greater for ankle muscles than for hip muscles and suggesting that visual reweighting differs across actuators on the short support surface. There were no phase differences between different surface conditions and also between two FRFs.

Figures 7C,D show the comparison between the direct-effect FRF from the vibration sensory perturbation to the weighted ankle EMG signal and the direct-effect FRF from the vibration sensory perturbation to the weighted hip EMG signal. The gain ratio of the direct-effect FRF from vibration to ankle EMG from 3rd to 6th frequency bin was higher than 1. The gain ratio of the direct-effect FRF from vibration to hip EMG in the 8th frequency bin was lower than 1. In addition, there was a significant difference between two direct-effect FRFs at the 8th frequency bin (*p* = 0.03). This means subjects downweighted proprioception for ankle muscles and hip muscles around 2 Hz whereas, upweighted vision was constant with increasing frequency. Additionally, the downweighting was greater for the hip muscles than for the ankle muscles only at 2 Hz. However, there was no significant difference between means across 10 frequency bins of the gain ratio of two FRFs from vibration perturbations to ankle EMG and hip EMG. There also were no phase differences between different surface conditions and also between two FRFs.

## Discussion

The aim of this study was to decompose the control loop for human upright stance and isolate contributions of particular processes to behavioral change associated with standing on different support surfaces. By separately identifying the musculoskeletal and feedback components of the human postural control loop, as well as the “direct effect” of sensory perturbations, we found a dramatic phase reversal between visual input and body kinematics due to the change in surface condition. Decomposition indicated that the phase reversal is due to contributions from the plant and the direct effect of visual input, with no change in overall feedback.

### Decomposition of the control loop

We found a striking difference in the phase of the closed-loop kinematic responses to the visual perturbation between the two surface conditions (Figures 5A,B). The leg segment response began to lag behind the trunk's response on the normal support surface as frequency of visual perturbation increased. In contrast, the trunk segment response on the short support surface lagged behind the legs as frequency of visual perturbation increased. This phase reversal could be due to any combination of three components: plant, feedback and the direct effect. First, the plant may be different between the two conditions, that is, the same muscle activation pattern may produce a different kinematic response because of the mechanical differences of standing on a short surface. Second, feedback (i.e., neural feedback component of the control loop) may be different, because, for example, a subject adopts a different control strategy on a short surface. Third, the direct effect of the visual perturbation may be different, because the nervous system may re-weight the use of visual information on a short surface. Because we were able to separately identify the plant, feedback and direct sensory effect for each of the two surface conditions, we could evaluate the contribution of each component to the observed phase reversal between conditions (Figure 6). This evaluation showed that the phase reversal was largely due to condition differences in the plant and the direct effect of visual-scene motion.

Thus, our results suggest that the phase reversal between surface conditions is largely due to the difference in the mechanical interactions between the feet and the support surface (a difference in the plant) and sensory reweighting (a difference in the direct sensory effect). A full understanding of how plant and direct-effect differences contribute to the phase reversal would require mathematical modeling, especially because these contributions depend on the properties of neural feedback (Equation 4). However, some insight into the contribution of the direct effect follows from decomposing the direct effect into components mediated by the ankle EMG and hip EMG signals [Equation 5]. If these components have different relative phases between leg and trunk responses, then sensory reweighting that differentially affects ankle EMG and hip EMG (Figure 7), will change the relative phase between leg and trunk responses.

### Differential sensory reweighting of the direct effect

Sensory reweighting is the process through which the nervous system changes the “emphasis” of a particular sensory input due to neurological injury or when environmental conditions change. Sensory reweighting is typically characterized through changes in gain (sway amplitude divided by sensory input amplitude; Hwang et al., 2014). Recent studies suggested that the source of sensory information used for torque generation depends upon environmental conditions and the reliability of sensory orientation and movement information (Peterka, 2002; Peterka and Loughlin, 2004). They measured the stimulus-dependent changes in sensory contributions to postural control. Their results provide estimates of important postural control parameters (stiffness, damping, time delay) and demonstrated how these parameters change under different sensory stimulus conditions. However, they could not determine how sensory reweighting influenced muscle activation. By identifying FRFs for each sensory perturbation relative to each muscle, we found that visual upweighting is greater for ankle EMG than for hip EMG, suggesting that sensory reweighting differs across actuators (i.e., muscles) when the subject stands on short support surface. This emphasizes the interplay between sensory input and muscle activation for postural control. Such results may provide an avenue for treatment by developing sensory reweighting processes to enhance balance ability.

### Limitations of our system identification approach

This study belongs to a group of studies that uses the JIO method to study postural control (e.g., Fitzpatrick et al., 1996; Kiemel et al., 2008, 2011; Boonstra et al., 2013; Engelhart et al., 2015). The JIO method provides insight into the postural control system by non-parametrically identifying its components. However, this advantage of the JIO approach rests on two assumptions. First, the postural control system is assumed to be approximately linear in each condition, although certain non-linearities such sensory reweighting can be addressed by applying the JIO method separately to each condition. Second, one must approximate inputs to the plant and feedback using a small number of signals, since one perturbation is required for each input. To identify neural feedback, this means that movement of the body is approximated using a small number of mechanical degrees of freedom. The initial JIO study of Fitzpatrick et al. (1996) used a single degree of freedom (an ankle joint), resulting in identified feedback that was inconsistent with the presence of a feedback delay. Adding a second degree of freedom (a hip joint) solved this problem and yielded a plausible estimate of the neural feedback delay (Kiemel et al., 2011). More recent studies (Boonstra et al., 2013; Engelhart et al., 2015) have also use this two-joint (ankle and hip) approximation of body, which can be viewed as compromise between accuracy and experimental feasibility.

## Conclusions

System identification techniques have been used previously, but primarily to study the behavior of a stable plant, such as reaching (Lacquaniti et al., 1993). Starting with Fitzpatrick et al. (1996), the JIO method has been used to infer open-loop properties for human upright stance, with more recent studies emphasizing the importance of multi-joint body mechanics (Kiemel et al., 2008, 2011; Boonstra et al., 2013; Engelhart et al., 2015). For example, Kiemel et al. (2011) challenged the dogma that the nervous system tries to minimize sway during standing. Instead, minimization of muscle activity (i.e., neural effort) is critical. This is an example of how the JIO method uncovers important properties that are not discernible from the closed-loop responses.

To understand how different support surfaces change the closed-loop postural responses to sensory perturbations, we isolated contributions of the open-loop plant, neural feedback, and direct sensory effect. We found that the change in surface condition led to a dramatic phase reversal between leg and trunk responses to visual-scene motion. Additionally, decomposition showed that the phase reversal is largely due to contributions from the plant and the direct effect of visual perturbations. The change from a normal to short surface in the direct effect was due to visual upweighting being greater for the ankle EMG than for hip EMG, suggesting that sensory reweighting differs across actuators (i.e., muscles) when the subject stands on a short support surface. Such results demonstrate how closed-loop system identification techniques unravel how different processes within the control loop interact to stabilize the postural control system.

## Author contributions

SH, TK, and JJ conceived and designed the experiments. SH and PA performed the experiments. SH, PA, and TK analyzed the data. SH, TK, and JJ contributed reagents/materials/analysis tools. SH, TK, and JJ wrote the paper.

### Conflict of interest statement

The authors declare that the research was conducted in the absence of any commercial or financial relationships that could be construed as a potential conflict of interest.

## References

[B1] Bendat J. S. Piersol A. G. (2000). Random Data: Analysis and Measurement Procedures. New York, NY:Wiley.

[B2] Boonstra T. A. Schouten A. C. van der Kooij H. (2013). Identification of the contribution of the ankle and hip joints to multi-segmental balance control. J. Neuroeng. Rehabil. 10:23. 10.1186/1743-0003-10-23 23433148 PMC3662596

[B3] Boonstra T. A. Schouten A. C. van Vugt J. P. P. Bloem B. R. van der Kooij H. (2014). Parkinson's disease patients compensate for balance control asymmetry. J. Neurophysiol. 112, 3227–3239. 10.1152/jn.00813.2013 25253475

[B4] Creath R. Kiemel T. Horak F. Peterka R. Jeka J. (2005). A unified view of quiet and perturbed stance: simultaneous co-existing excitable modes. Neurosci. Lett. 377, 75–80. 10.1016/j.neulet.2004.11.071 15740840

[B5] Engelhart D. Pasma J. H. Schouten A. C. Meskers C. G. M. Maier A. B. Mergner T.. (2014). Impaired standing balance in elderly: a new engineering method helps to unravel causes and effects. J. Am. Med. Dir. Assoc. 15, 227.e1–227.e6. 10.1016/j.jamda.2013.09.009 24220138

[B6] Engelhart D. Schouten A. C. Aarts R. G. K. M. van der Kooij H. (2015). Assessment of multi-joint coordination and adaptation in standing balance: a novel Device and system identification technique. IEEE Trans. Neural Syst. Rehab. Eng. 23, 973–982. 10.1109/TNSRE.2014.2372172 25423654

[B7] Fitzpatrick R. C. Burke D. Gandevia S. C. (1996). Loop gain of reflexes controlling human standing measured with the use of postural and vestibualr disturbances. J. Neurophysiol. 76, 3994–4008. 8985895 10.1152/jn.1996.76.6.3994

[B8] Hall P. (1988). Theoretical comparison of bootstrap confidence-intervals. Ann. Stat. 16, 927–953. 10.1214/aos/1176350933

[B9] Horak F. B. Nashner L. M. (1986). Central programming of postural movements: adaptation to altered support-surface configurations. J. Neurophysiol. 55, 1369–1381. 3734861 10.1152/jn.1986.55.6.1369

[B10] Houk J. C. Rymer W. Z. (1981). Neural control of muscle length and tension, in Handbook of Physiology—The Nervous System II, eds Brookhart J. M. Mountcastle V. B. Brooks V. B. Geiger S. R. (Bethesda, MD: V. B. Brooks), 257–323.

[B11] Hwang S. Agada P. Kiemel T. Jeka J. J. (2014). Dynamic reweighting of three modalities for sensor fusion. PLoS ONE 9:e88132. 10.1371/journal.pone.0088132 24498252 PMC3909337

[B12] Katayama T. (2005). Subspace Methods for System Identification. London: Springer.

[B13] Kiemel T. Elahi A. J. Jeka J. J. (2008). Identification of the plant for upright stance in humans: multiple movement patterns from a single neural strategy. J. Neurophysiol. 100, 3394–3406. 10.1152/jn.01272.2007 18829854 PMC2604857

[B14] Kiemel T. Zhang Y. F. Jeka J. J. (2011). Identification of neural feedback for upright stance in humans: stabilization rather than sway minimization. J. Neurosci. 31, 15144–15153. 10.1523/JNEUROSCI.1013-11.2011 22016548 PMC3470452

[B15] Lacquaniti F. Corrozzo M. Borghese N. A. (1993). The role of vision in tuning the anticipatory motor responses of the limbs, in Multisensory Control of Movement, ed Berthoz A. (New York, NY: Oxford), 379–393.

[B16] Leigh R. J. Newman S. A. Zee D. S. Miller N. R. (1982). Visual following during stimulation of an immobile eye (the open loop condition). Vision Res. 22, 1193–1197. 10.1016/0042-6989(82)90084-0 6983180

[B17] McIlroy W. E. Maki B. E. (1997). Preferred placement of the feet during quiet stance: development of a standardized foot placement for balance testing. Clin. Biomech. 12, 66–70. 10.1016/S0268-0033(96)00040-X 11415674

[B18] Pasma J. H. Engelhart D. Schouten A. C. van der Kooij H. Maier A. B. Meskers C. G. M. (2014). Impaired standing balance: the clinical need for closing the loop. Neuroscience 267, 157–165. 10.1016/j.neuroscience.2014.02.030 24613719

[B19] Peterka R. J. (2002). Sensorimotor integration in human postural control. J Neurophysiol. 88, 1097–1118. 10.1152/jn.00605.2001 12205132

[B20] Peterka R. J. Black F. O. (1990). Age-related changes in human posture control: sensory organization tests. J. Vestib. Res. 1, 73–85. 1670139

[B21] Peterka R. J. Loughlin P. J. (2004). Dynamic regulation of sensorimotor integration in human postural control. J. Neurophysiol. 91, 410–423. 10.1152/jn.00516.2003 13679407

[B22] Stark L. W. (1984). The pupil as a paradigm for neurological control systems. IEEE Trans. Biomed. Eng. 31, 919–924. 10.1109/TBME.1984.325259 6396219

[B23] van der Kooij H. Jacobs R. Koopman B. van der Helm F. (2001). An adaptive model of sensory integration in a dynamic environment applied to human stance control. Biol. Cybern. 84, 103–115. 10.1007/s004220000196 11205347

[B24] van der Kooij H. van Asseldonk E. Geelen J. van Vugt J. P. Bloem B. R. (2007). Detecting asymmetries in balance control with system identification: first experimental results from Parkinson patients. J. Neural Transm. 114, 1333–1337. 10.1007/s00702-007-0801-x 17703275

[B25] van der Kooij H. van Asseldonk E. van der Helm F. C. (2005). Comparison of different methods to identify and quantify balance control. J. Neurosci. Methods 145, 175–203. 10.1016/j.jneumeth.2005.01.003 15922036

[B26] Zoubir A. M. Boashash B. (1998). The bootstrap and its application in signal processing. IEEE Signal Process. Mag. 15, 56–76. 10.1109/79.647043

