# Supplementary material for: Identification of the Unstable Human Postural Control System
Source: Front Syst Neurosci. 2016 Mar 11;10:22. doi: 10.3389/fnsys.2016.00022 (PMC4786559; doi:10.3389/fnsys.2016.00022)
Supplement: Supplementary file 1 [file Presentation1.PDF]

Supplementary Material

Identification of the unstable human postural control system

Sungjae Hwang*, Peter Agada, Tim Kiemel, John J. Jeka

*** Correspondence:** Sungjae Hwang: sungjae.hwang@temple.edu

1. Decomposition of human postural control loop

First of all, closed-loop FRFs from sensory perturbations to weighted EMG signals and body segment angles were used to identify the open-loop FRF describing the plant and the open-loop FRF characterizing the direct effect of sensory perturbations on EMG signals.

Supplementary Figure 1 shows FRFs for the plant on a normal support surface. Supplementary Figure 1A-B shows the gains and phases of the mean closed-loop FRF $\overline{H}_{vu}(f)$ from the vision and the vibration, which is for the stimulation to the proprioception, sensory perturbations to the weighted ankle and hip EMG signal. Supplementary Figure 1C-D shows the gains and phases of the mean closed-loop FRF $\overline{H}_{vy}(f)$ from the vision and the vibration sensory perturbation to the leg and trunk segment angle. Supplementary Figure 1E-F shows the gains and phases of the mean open-loop FRF $P(f)$ describing the mapping from the weighted ankle and hip EMG signal to the leg and trunk segment angle. Figure 1G-H shows the gains and phases of the mean open-loop FRF $S(f)$ describing the mapping from the vision and the vibration sensory perturbation to the weighted ankle and hip EMG signal. Supplementary Figure 2 shows FRFs for the plant on a short support surface. Supplementary Figure 2A-H shows the gains and phases in the same way as Supplementary Figure 1A-H.

It is difficult to mechanistically interpret the closed-loop FRFs $\overline{H}_{vu}(f)$ and $\overline{H}_{vy}(f)$, because they reflect the closed-loop interaction between the plant and feedback components of the postural control feedback loop as well as the properties of the sensory perturbations. However, the relationship between the two closed-loop FRFs depends only on the plant component. Specifically, the inferred open-loop FRF of the plant is $P(f) = H_{vy}(f)H_{vu}(f)^{-1}$, whose gain and phase are shown in Supplementary Figure 1E-F and Supplementary Figure 2E-F. The plant is the mapping from weighted ankle and hip EMG signals to the leg and trunk segment angles. Gains eventually decreased with increasing frequency, indicating that the plant acts as a low-pass filter in response to muscle activation. This pattern from the experimental results was agreed with our previous simulation results using a mechanistic plant model (Kiemel et al., 2011). Phase for the plant indicated that at low frequencies, activation of posterior ankle muscles produced a backward rotation of the legs as ankle plantarflexion and a forward rotation of the trunk as hip flexion, whereas activation of posterior hip

muscles produced a forward rotation of the legs as ankle dorsiflexion and a backward rotation of the trunk as hip extension.

In $\overline{H}_{vu}(f)$, there were significant gain differences between conditions in FRF which is mapping from the vision sensory perturbation to the ankle EMG (blue filled circles in Supplementary Figure 1A and Supplementary Figure 2A) (p<0.0001), FRF from the vibration sensory perturbation to the ankle EMG (red unfilled circles in Supplementary Figure 1A and Supplementary Figure 2A) (p=0.01), and FRF from the vibration sensory perturbation to the hip EMG (red unfilled triangles in Supplementary Figure 1A and Supplementary Figure 2A) (p<0.0001). Significant phase differences between conditions were in FRF from the vision sensory perturbation to the ankle EMG (blue filled circles in Supplementary Figure 1B and Supplementary Figure 2B) (p<0.001) and FRF from the vision sensory perturbation to the hip EMG (blue filled triangles in Supplementary Figure 1B and Supplementary Figure 2B).

In $\overline{H}_{vy}(f)$, there were significant gain differences between conditions in FRF which is mapping from the vision sensory perturbation to the legs segment angle (blue filled circles in Supplementary Figure 1C and Supplementary Figure 2C) (p=0.02), FRF from the vision sensory perturbation to the trunk segment angle (red unfilled circles in Supplementary Figure 1C and Supplementary Figure 2C) (p=0.004), FRF from the vibration sensory perturbation to the legs segment angle (blue filled triangles in Supplementary Figure 1C and Supplementary Figure 2C) (p<0.0001), and FRF from the vibration sensory perturbation to the trunk segment angle (red unfilled triangles in Supplementary Figure 1C and Supplementary Figure 2C) (p<0.0001). Significant phase differences between conditions were in FRF from the vision sensory perturbation to the legs segment angle (blue filled circles in Supplementary Figure 1D and Supplementary Figure 2D) (p<0.001), FRF from the vision sensory perturbation to the trunk segment angle (red unfilled circles in Supplementary Figure 1D and Supplementary Figure 2D) (p<0.001), and FRF from the vibration sensory perturbation to the legs segment angle (blue filled triangles in Supplementary Figure 1D and Supplementary Figure 2D) (p<0.01).

In $P(f)$, there were no significant gain differences (Supplementary Figure 1E and Supplementary Figure 2E) as well as phase differences (Supplementary Figure 1F and Supplementary Figure 2F) between conditions. In $S(f)$, there were significant gain differences between conditions in FRF which is mapping from the vision sensory perturbation to the ankle EMG (blue filled circles in Supplementary Figure 1G and Supplementary Figure 2G) (p<0.0001), FRF from the vision sensory perturbation to the hip EMG (blue filled triangles in Supplementary Figure 1G and Supplementary Figure 2G) (p<0.0001), and FRF from the vibration sensory perturbation to the ankle EMG (red unfilled circles in Supplementary Figure 1G and Supplementary Figure 2G) (p=0.03). There are no significant phase differences between conditions (Supplementary Figure 1H and Supplementary Figure 2H).

The most remarkable difference between the normal support surface and the short support surface occurred in the phase of the closed-loop FRF from the vision perturbation to the leg and trunk segment angels (Figure Supplementary 1D and Supplementary Figure 2D). On the normal support surface, as frequency of visual perturbation increases, larger negative phase values indicate that the legs' response begins to lag behind the trunk's response (i.e., trunk leads legs), as shown in

Supplementary Figure 1D. In contrast, on the short support surface it is the trunk that lags behind the legs (i.e., legs lead trunk), as shown in Supplementary Figure 2D.

Secondly, closed-loop FRFs from mechanical perturbations to weighted EMG signals and body segment angles were used to identify the open-loop FRF describing the feedback and the open-loop FRF characterizing the effect of mechanical perturbations to body segment angles.

Supplementary Figure 3 shows FRFs for the feedback on a normal support surface. Supplementary Figure 3A-B shows the gains and phases of the mean closed-loop FRF $\overline{H}_{du}(f)$ from the waist and shoulder mechanical perturbation to the weighted ankle and hip EMG signal. Supplementary Figure 3C-D shows the gains and phases of the mean closed-loop FRF $\overline{H}_{dy}(f)$ from the waist and shoulder mechanical perturbation to the leg and trunk segment angle. Supplementary Figure 3E-F shows the gains and phases of the mean open-loop FRF $F(f)$ describing the mapping from the leg and trunk segment angle to the weighted ankle and hip EMG signal. Supplementary Figure 3G-H shows the gains and phases of the mean open-loop FRF $M(f)$ describing the mapping from the waist and shoulder mechanical perturbation to the leg and trunk segment angle. Supplementary Figure 4 shows FRFs for the feedback on a short support surface. Supplementary Figure 4A-H shows the gains and phases in the same way as Supplementary Figure 3A-H.

Similarly, it is difficult to mechanistically interpret the closed-loop FRFs $\overline{H}_{du}(f)$ and $\overline{H}_{dy}(f)$, because they reflect the closed-loop interaction between the plant and feedback components of the postural control feedback loop as well as the properties of the mechanical perturbations. However, the relationship between the two closed-loop FRFs depends only on the feedback component. Specifically, the inferred open-loop FRF of the feedback is $F(f) = H_{du}(f)H_{dy}(f)^{-1}$, whose gain and phase are shown in Supplementary Figure 3E-F and Supplementary Figure 4E-F. The feedback is the mapping from leg and trunk segment angle to weighted ankle and hip EMG signal. The gain of the feedback is roughly constant (Supplementary Figure 3E) and phase is near 0 (Supplementary Figure 3F) at low frequencies. This indicated that the activations of ankle and hip muscles are roughly proportional to deviations of segment angles from their mean positions. As frequency increases, gains increase and phases also initially increase. This pattern is consistent with feedback that also depends on segment-angle velocities, that is, proportional-derivative (PD) control (Peterka, 2002; Fitzpatrick et al., 1996; Johansson et al., 1988). With increasing frequency, phase reach a maximum between 71 and 81 at the 6th and 7th frequency bins and then decrease. The eventual decrease in phase is consistent with results of previous studies showing the phase decrease of the optimal feedback model at higher frequencies that would be expected given a feedback time delay (van der Kooij et al., 2005; Kiemel et al., 2011).

In $\overline{H}_{du}(f)$, there was a significant gain difference between conditions in FRF which is mapping from the shoulder mechanical perturbation to the hip EMG (red unfilled triangles in Supplementary Figure 3A and Supplementary Figure 4A) (p=0.01). Significant phase differences between conditions were in FRF from the waist mechanical perturbation to the ankle EMG (blue filled circles in Supplementary Figure 3B and Supplementary Figure 4B) ($p<0.01$) and FRF from the

waist mechanical perturbation to the hip EMG (blue filled triangles in Supplementary Figure 3B and Supplementary Figure 4B) ($p<0.05$).

In $\overline{H}_{dy}(f)$, there were significant gain differences between conditions in FRF which is mapping from the waist mechanical perturbation to legs segment angle (blue filled circles in Supplementary Figure 3C and Supplementary Figure 4C) ($p<0.0001$) and FRF from the waist mechanical perturbation to the trunk segment angle (red unfilled circles in Supplementary Figure 3C and Supplementary Figure 4C) ($p<0.0001$). Significant phase differences between conditions were in FRF which is mapping from the waist mechanical perturbation to legs segment angle (blue filled circles in Supplementary Figure 3D and Supplementary Figure 4D) ($p<0.01$) and FRF from the shoulder mechanical perturbation to legs segment angle (blue filled triangles in Supplementary Figure 3D and Supplementary Figure 4D) ($p<0.01$).

In $F(f)$, there were significant gain differences between conditions in FRF which is mapping from the legs segment angle to the ankle EMG (blue filled circles in Supplementary Figure 3E and Supplementary Figure 4E) ($p=0.0006$), FRF from the trunk segment angle to the ankle EMG (red unfilled circles in Supplementary Figure 3E and Supplementary Figure 4E) ($p=0.001$), and FRF from the trunk segment angle to the hip EMG (red unfilled triangles in Supplementary Figure 3E and Supplementary Figure 4E) ($p=0.01$). There are no significant phase differences between conditions (Supplementary Figure 3F and Supplementary Figure 4F).

In $M(f)$, there was a significant gain difference between conditions in FRF which is mapping from the waist mechanical perturbation to legs segment angle (blue filled circles in Supplementary Figure 3G and Supplementary Figure 4G) ($p<0.0001$). There are no significant phase differences between conditions (Supplementary Figure 3H and Supplementary Figure 4H).

Overall patterns of all FRFs of the feedback were similar and we couldn’t find any remarkable difference of the feedback between the normal support surface and the short support surface.

1.1 Supplementary Figures

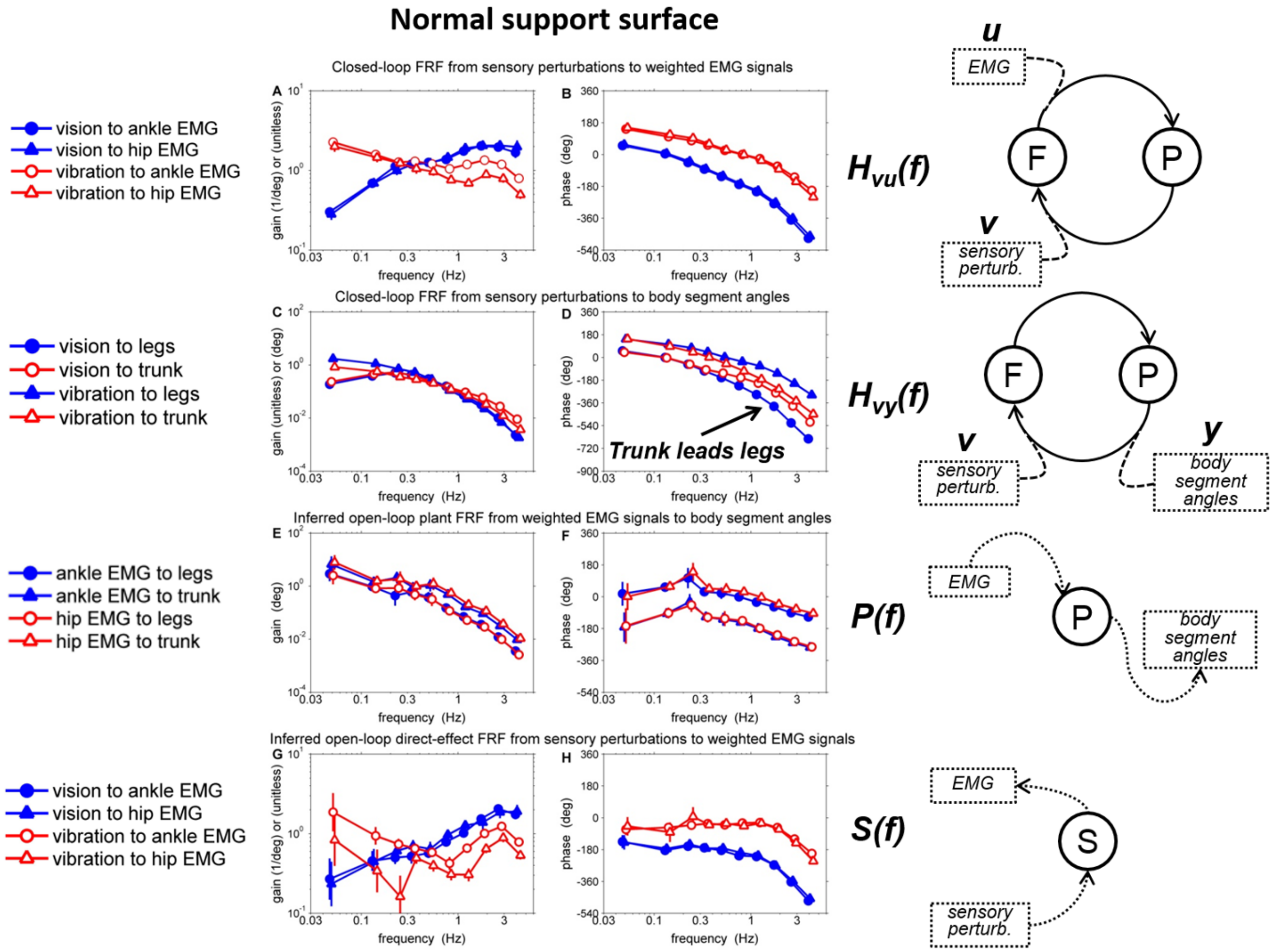

Supplementary Figure 1. FRFs of the plant on a normal support surface. (A) gain of closed-loop FRF from sensory perturbations to weighted EMG signals (B) phase of closed-loop FRF from sensory perturbations to weighted EMG signals (C) gain of closed-loop FRF from sensory perturbations to body segment angels (D) phase of closed-loop FRF from sensory perturbations to body segment angels (E) gain of inferred open-loop plant FRF from weighted EMG signals to body segment angles (F) phase of inferred open-loop plant FRF from weighted EMG signals to body segment angles (G) gain of inferred open-loop direct-FRF from sensory perturbations to weighted EMG signals (H) phase of inferred open-loop direct-FRF from sensory perturbations to weighted EMG signals. Error bars denote bootstrap standard errors.

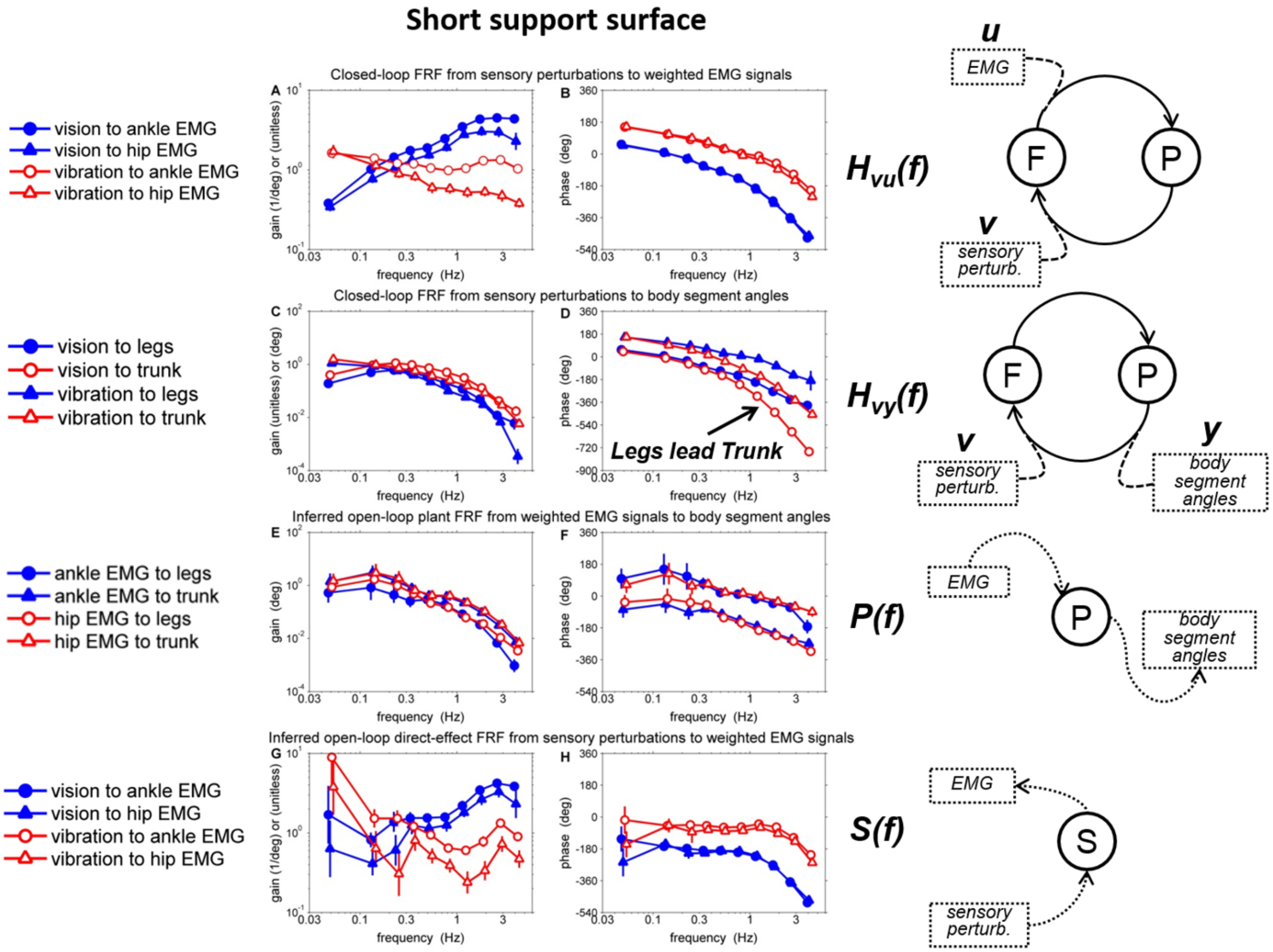

Supplementary Figure 2. FRFs of the plant on a short support surface. (A) gain of closed-loop FRF from sensory perturbations to weighted EMG signals (B) phase of closed-loop FRF from sensory perturbations to weighted EMG signals (C) gain of closed-loop FRF from sensory perturbations to body segment angels (D) phase of closed-loop FRF from sensory perturbations to body segment angels (E) gain of inferred open-loop plant FRF from weighted EMG signals to body segment angles (F) phase of inferred open-loop plant FRF from weighted EMG signals to body segment angles (G) gain of inferred open-loop direct-FRF from sensory perturbations to weighted EMG signals (H) phase of inferred open-loop direct-FRF from sensory perturbations to weighted EMG signals. Error bars denote bootstrap standard errors.

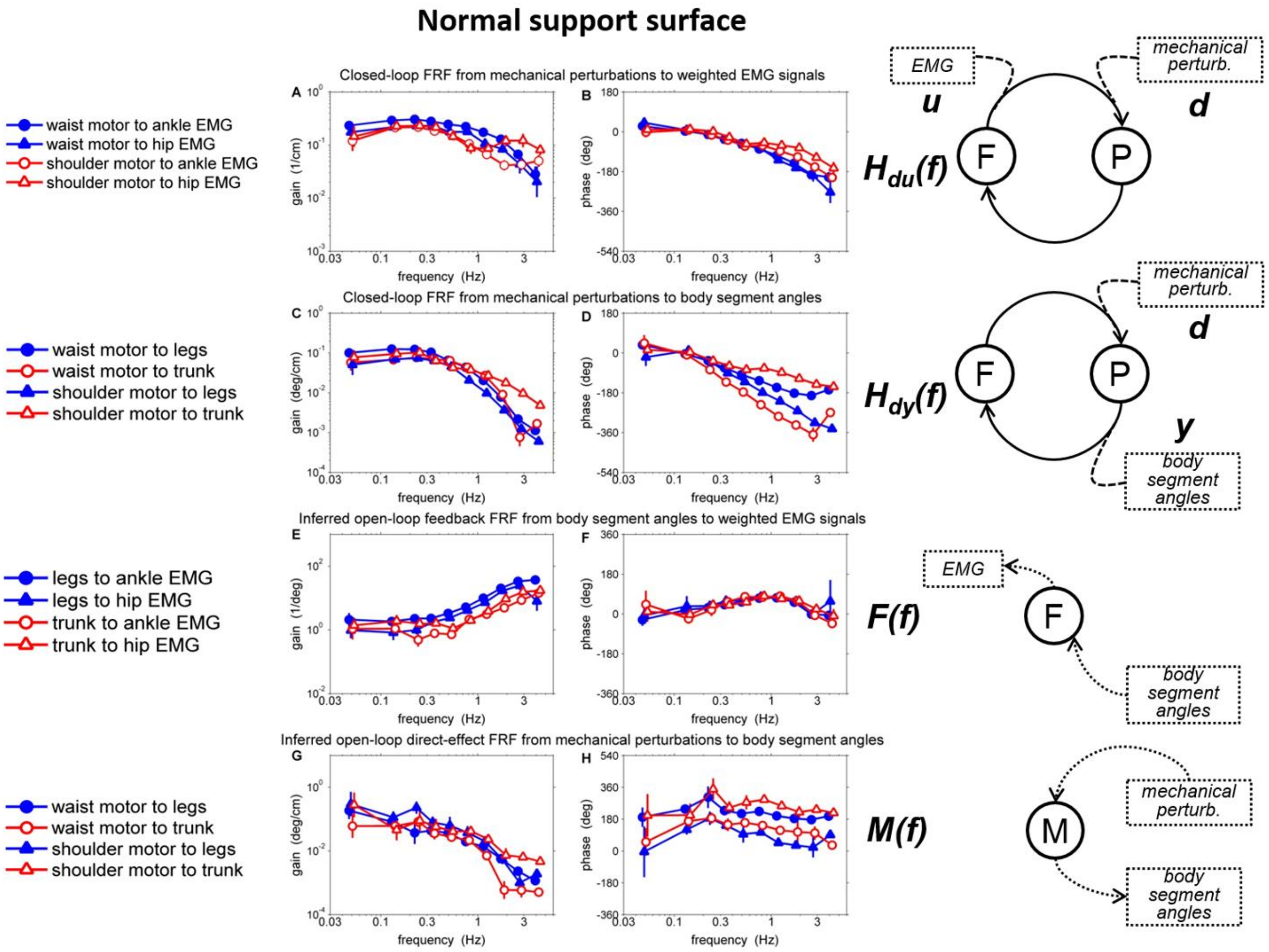

Supplementary Figure 3. FRFs of the feedback on a normal support surface. (A) gain of closed-loop FRF from mechanical perturbations to weighted EMG signals (B) phase of closed-loop FRF from mechanical perturbations to weighted EMG signals (C) gain of closed-loop FRF from mechanical perturbations to body segment angels (D) phase of closed-loop FRF from mechanical perturbations to body segment angels (E) gain of inferred open-loop feedback FRF from body segment angles to weighted EMG signals (F) phase of inferred open-loop feedback FRF from body segment angles to weighted EMG signals (G) gain of inferred open-loop direct-FRF from mechanical perturbations to body segment angels (H) phase of inferred open-loop direct-FRF from mechanical perturbations to body segment angels. Error bars denote bootstrap standard errors.

Supplementary Figure 4.

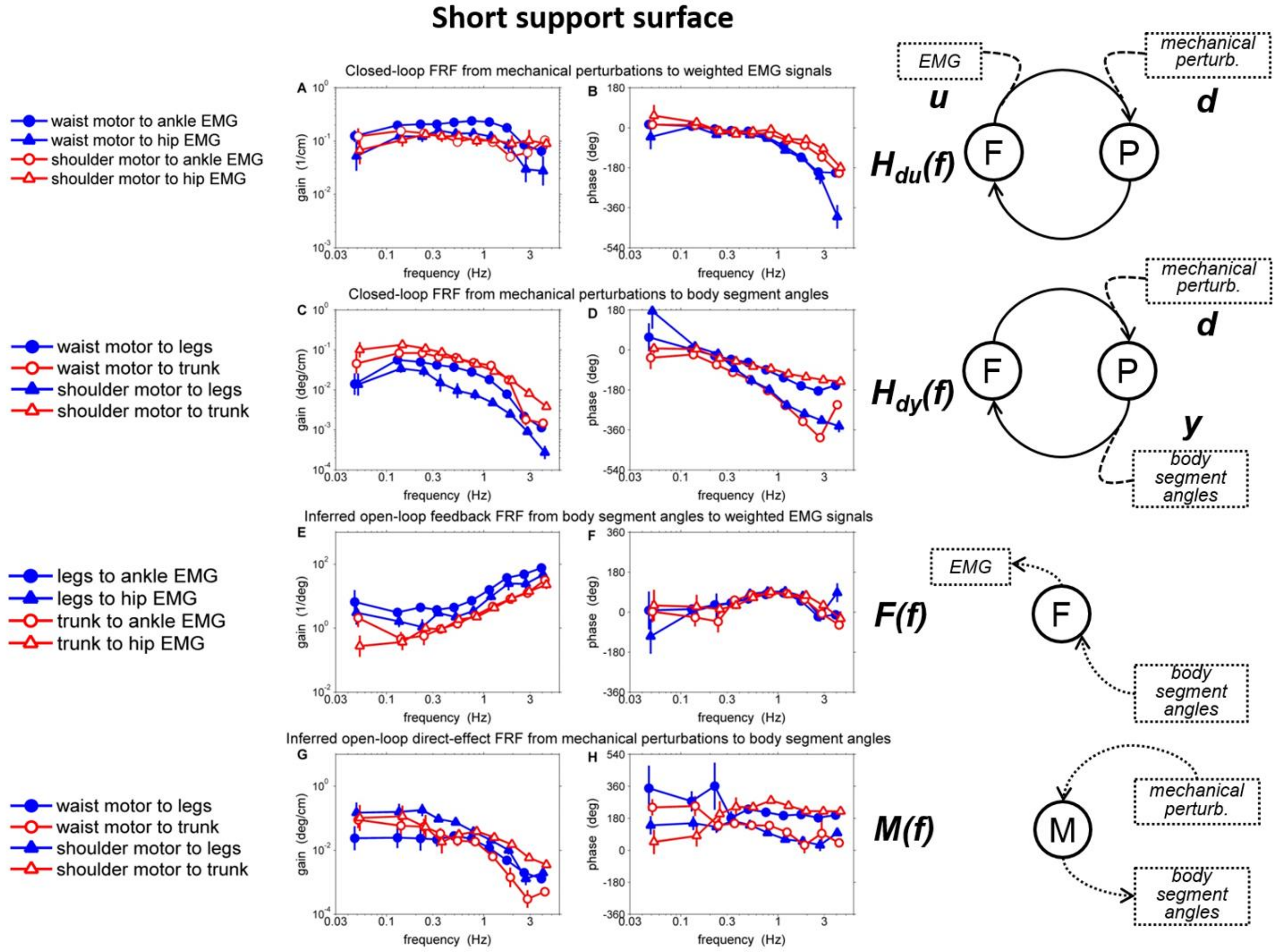

Supplementary Figure 4. FRFs of the feedback on a short support surface. (A) gain of closed-loop FRF from mechanical perturbations to weighted EMG signals (B) phase of closed-loop FRF from mechanical perturbations to weighted EMG signals (C) gain of closed-loop FRF from mechanical perturbations to body segment angels (D) phase of closed-loop FRF from mechanical perturbations to body segment angels (E) gain of inferred open-loop feedback FRF from body segment angles to weighted EMG signals (F) phase of inferred open-loop feedback FRF from body segment angles to weighted EMG signals (G) gain of inferred open-loop direct-FRF from mechanical perturbations to body segment angels (H) phase of inferred open-loop direct-FRF from mechanical perturbations to body segment angels. Error bars denote bootstrap standard errors.
