# Supplementary figures and images for: Identification of the Unstable Human Postural Control System
Source: Front Syst Neurosci. 2016 Mar 11;10:22. doi: 10.3389/fnsys.2016.00022 (PMC4786559; doi:10.3389/fnsys.2016.00022)

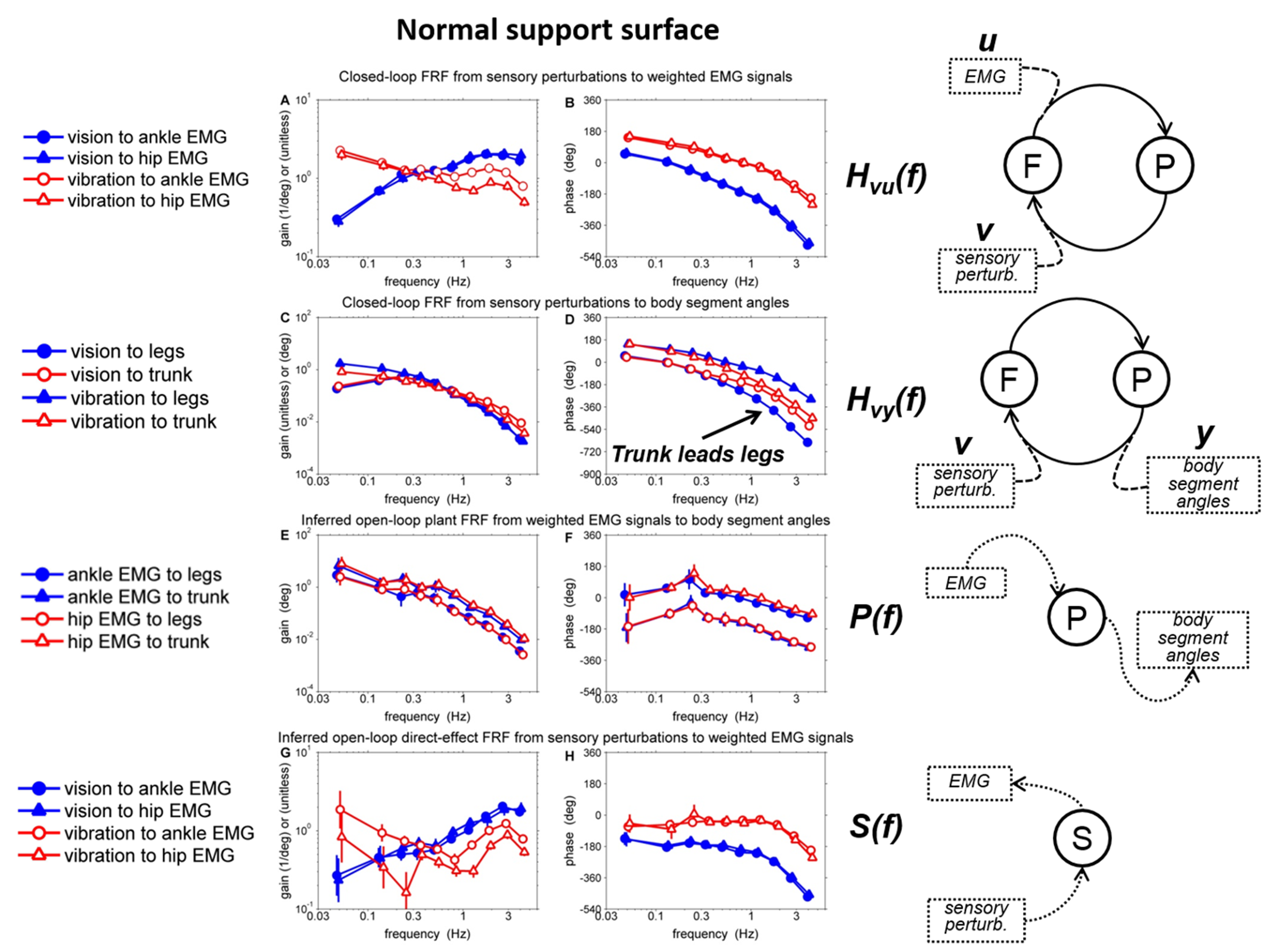

Supplement: Supplementary file 2 [file Image1.TIF]

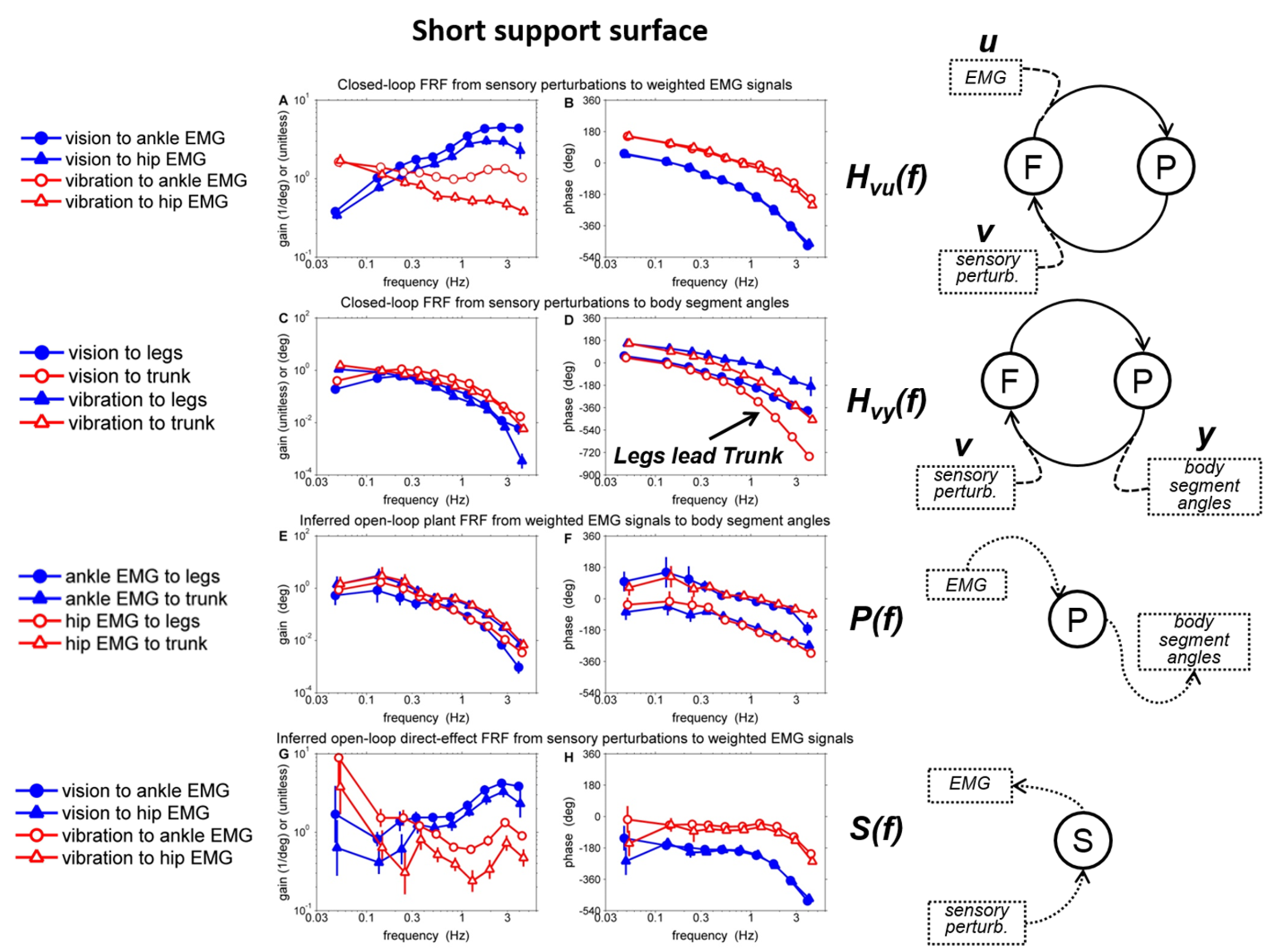

Supplement: Supplementary file 3 [file Image2.TIF]

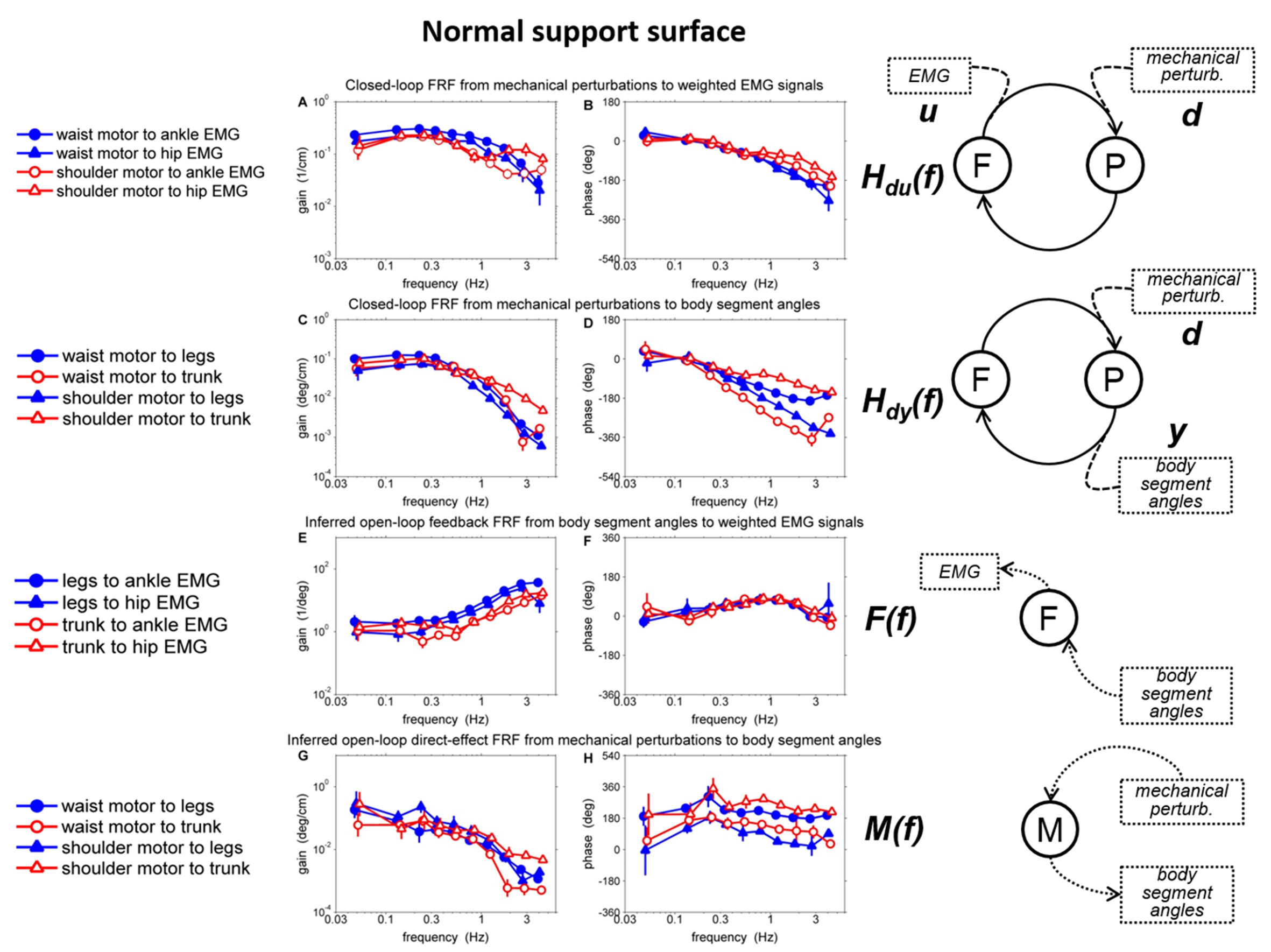

Supplement: Supplementary file 4 [file Image3.TIF]

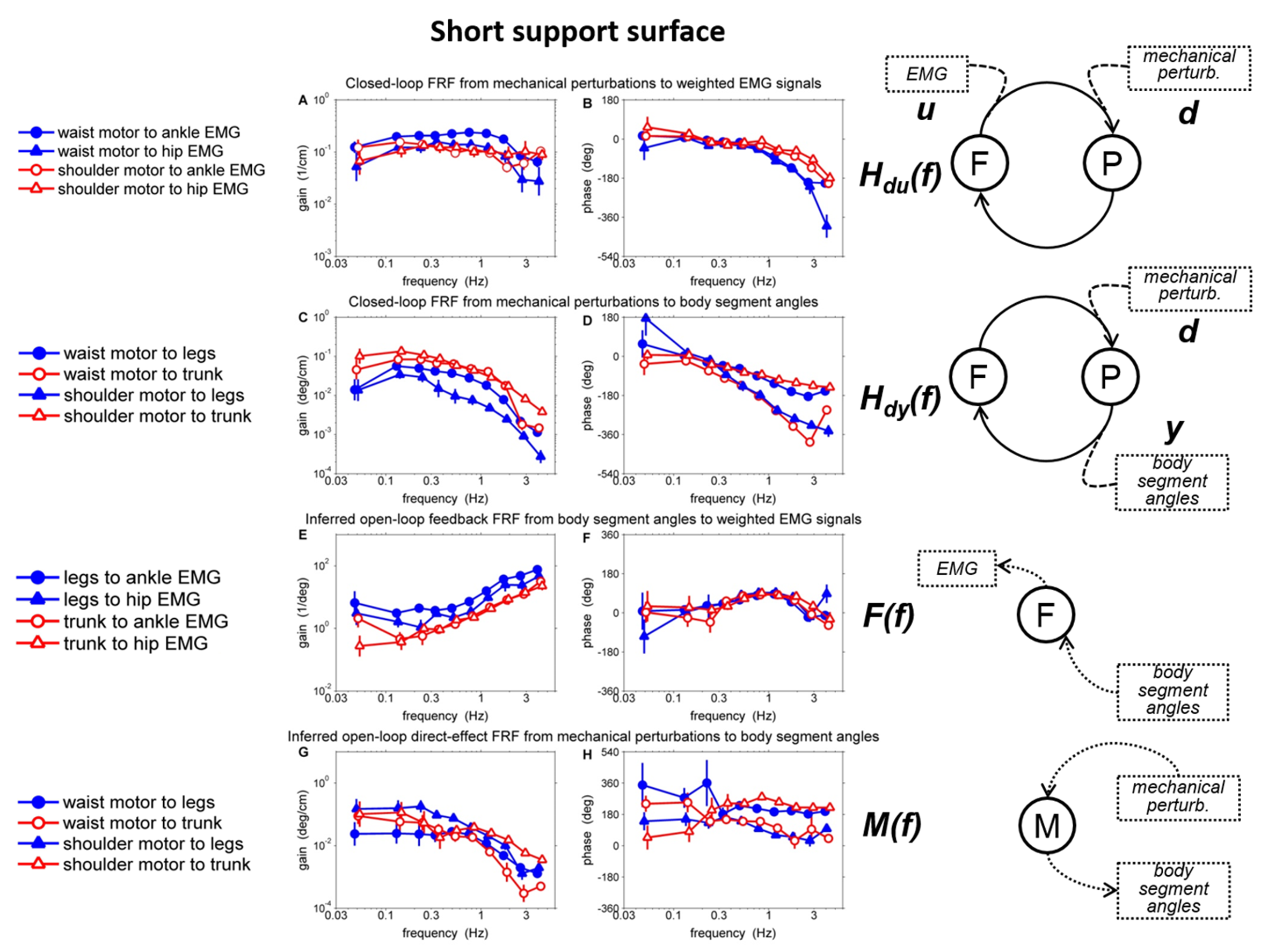

Supplement: Supplementary file 5 [file Image4.TIF]
